# Ancient DNA study provides clues to leprosy susceptibility in medieval Europe

**DOI:** 10.1186/s13059-025-03925-8

**Published:** 2026-01-16

**Authors:** Joanna H. Romeyer-Dherbey, Amke Caliebe, Onur Ӧzer, Nicolas Antonio da Silva, Nicolás Mendoza Mejía, Daniel Anton Myburgh, Katharina Fuchs, Dorthe Dangvard Pedersen, Jesper Boldsen, Lars Agersnap Larsen, Lone Seeberg, Morten Søvsø, Dirk Rieger, Andreas Prescher, Almut Nebel, Ben Krause-Kyora

**Affiliations:** 1 Institute of Clinical Molecular Biology, Kiel University, Rosalind-Franklin Straße 12, Kiel, 24105 Germany; 2 Institute of Medical Informatics and Statistics, Kiel University, Brunswiker Straße 10, Kiel, 24105 Germany; 3 Institute of Prehistoric and Protohistoric Archaeology, Kiel University, Johanna-Mestorf Straße 2, Kiel, 24118 Germany; 4 Unit of Anthropology, Department of Forensic Medicine, University of Southern Denmark, Odense M, 5230 Denmark; 5 Viborg Museum, Sct. Mogens Gade 5, Viborg, 8800 Denmark; 6 Museum Horsens, Fussingsvej 8, Horsens, 8700 Denmark; 7 Museum West, Tangevej 6B, Ribe, 6760 Denmark; 8 Department of Archaeology, Hanseatic City of Lübeck Historic Monuments Protection Authority, Königstraße 21, Lübeck, 23539 Germany; 9 Institute of Molecular and Cellular Anatomy, University Clinic RWTH Aachen, Wendlingweg 2, Aachen, 52074 Germany; 10 Current affiliation: Department of Genetics, Yale School of Medicine, New Haven, CT USA

**Keywords:** Ancient DNA, Ancient genomics, Human leukocyte antigens, Case–control association study, Human immunity, Leprosy, *Mycobacterium leprae*, Pathogen-driven selection, Middle Ages, Epidemics

## Abstract

**Background:**

Leprosy is a chronic infectious disease caused by *Mycobacterium leprae* (*M. leprae*) that reached an epidemic scale in the Middle Ages. Nowadays, the disease is absent in Europe and host genetic influences have been considered as a contributing factor to leprosy disappearance. In this study, we perform a case–control association analysis between multiple human leukocyte antigen (HLA) alleles and leprosy in a medieval European population. The sample comprises 302 individuals from 18 archaeological sites in Denmark (*N* = 16) and Germany (*N* = 2).

**Results:**

Our results indicate that HLA-B*38 is associated with leprosy risk. Furthermore, we detect three novel variants that were possibly involved in leprosy risk or protection: HLA-A*23, DRB1*04, and DRB1*13. We also note a subtle temporal change in frequency for several alleles previously associated with infectious diseases, inflammatory disorders, and cancer in present-day populations.

**Conclusions:**

This study demonstrates the potential of ancient DNA in the identification of genetic variants involved in predisposition to diseases that are no longer present in Europe but remain endemic elsewhere. Although it is difficult to pinpoint the reason behind the temporal frequency shift, past epidemics of infectious diseases have likely influenced the HLA pool in present-day Europe.

**Supplementary Information:**

The online version contains supplementary material available at 10.1186/s13059-025-03925-8.

## Background

Based on historical documentation, archaeological sources, and molecular data, it is evident that medieval Europeans struggled with a high disease burden and the ever-present threat of infections [e.g., [1–4]. One of the diseases that reached an epidemic scale in the Middle Ages was leprosy (Hansen’s disease) [5–7] caused by a *Mycobacterium leprae* (*M. leprae*) infection. Due to the preference of *M. leprae* for lower temperatures, leprosy mainly influences the superficial structures, such as skin, mucous membranes, eyes, testes, and peripheral nerves [8]. Blindness and sensory loss often lead to injuries and secondary infections, which can result in complete loss of extremities, making leprosy highly debilitating. As the disease progresses over time, it causes skeletal lesions, some of which are diagnostic of leprosy. This often allows retrospective diagnosis of advanced leprosy in past populations based on macroscopic analysis of skeletal remains. As nasal mucosa and extremities are the main foci for leprosy, bones of the underlying facial skeleton, hands, and feet are most often affected [9]. The clearly visible disfigurements of the face and hands have led to ostracism and subsequent isolation of leprosy-affected individuals from society. For this purpose, specialized institutions called leprosaria were established in the Middle Ages. As leprosy is a lifelong disease, leprosaria became a home for the sick who were eventually buried at the associated cemeteries [4, 10, 11]. The illness virtually disappeared from Europe in the 16^th^ century, with only a few cases being reported until the 19^th^ century [12–14]. The reason behind leprosy’s disappearance from Europe is unclear. Considering the high genomic conservation of *M. leprae* since the early Middle Ages [6, 15–18], it is likely that environmental and host genetic factors played a major role in the disappearance of the disease from the European continent [17].

Although the clinical outcome of an infection may depend on various intrinsic and extrinsic factors [19–22], leprosy is an epitome of the substantial influence of the host’s immune response on disease manifestation. Depending on the degree of the immunological reaction, *M. leprae* infection can lead to severe disability, mild disfigurement, painful chronic inflammation, or a complete lack of symptoms [23–25]. Several association studies have identified genetic variants conferring susceptibility to or protection against the disease for populations in which it is endemic today, such as India, China, Brazil, and Mexico [e.g., [26–30]. Many associations were reported for variants in the human leukocyte antigen (HLA) region (reviewed in [31, 32]). As Europe has been free of leprosy for centuries, all modern association studies have been carried out in non-European populations. So far, only one ancient DNA (aDNA) leprosy case–control investigation on medieval Europeans has been performed [17]. The study confirmed the association between HLA-DRB1*15:01 and leprosy risk that had been reported previously in present-day non-Europeans [33–37]. Apart from this finding, however, the effect of additional HLA variation on the leprosy risk of Europeans remains unexplored. Here, we conducted an HLA association study in a collective of 302 medieval Europeans (Danish and Germans). To this end, we first identified leprosy-affected (cases) and non-affected (controls) individuals based on a set of criteria including archaeological context of the burial, palaeopathological analysis of the skeletons, and screening for *M. leprae* DNA. Subsequently, we genotyped the samples for the six classical HLA loci (class I: HLA-A, B, C; class II: HLA-DRB1, DQB1, and DPB1) to detect genetic variants involved in leprosy susceptibility.

## Results

### Sample collection

**Fig. 1:**
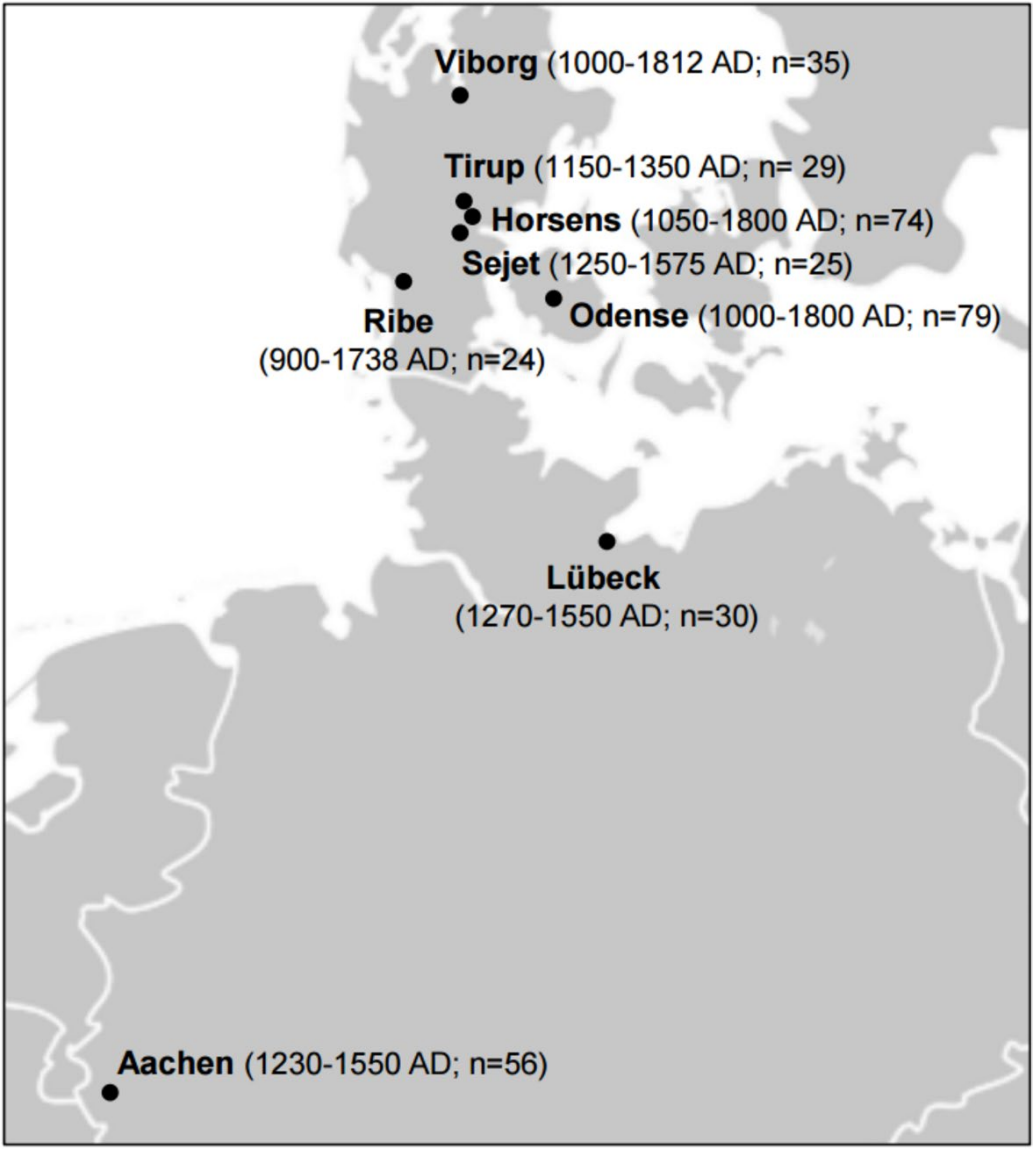
Map of archaeological sites. The number of individuals included in the study from each site is shown in brackets

Skeletal remains of 388 individuals excavated from 16 Danish and two German archaeological sites (10^th^-19^th^ century AD) were available. Of these, 352 individuals had sufficient endogenous DNA to be included in this study (Fig. 1; Table 1; Additional file 1: Skeletal_collections-literature). The cemeteries of Skt. Jørgensgården in Odense (Denmark) and Gut Melaten in Aachen (Germany) were associated with institutions that housed leprosy-affected individuals in the Middle Ages [38–40]. The remaining sites are ordinary cemeteries, except for a multiple burial at Albani Torv (Additional file 1: Skeletal_collections-literature). As the Klosterkirke burials postdate both leprosaria-associated cemeteries (Table 1), further analysis was performed to ensure that the individuals from this site could be included in the association study (see below).

**Table 1:** Number of examined individuals per archaeological site

Site	Location	Dating (year AD)	N
Skt. Jørgensgården^†^	Odense	1270–1550	68
Skt. Knuds Plads		1086–1800	2
Albani Torv		1000–1540	8
Klosterbakken		1086–1800	1
Skt. Trinitatis/Drotten	Viborg	1000–1529	13
Skt. Morten		1000–1529	4
Skt. Mathias		1000–1529	2
Skt. Mikkel		1000–1800	11
Gråbrødrekloster		1529–1812	2
Faldborg Kirkegård		1100-mid 16^th^ c.	3
Klosterkirke	Horsens	1600–1800	50
Ole Worms Gade		1050–1536	24
Ødekirkegård	Sejet	1250–1575	25
Tirup kirke	Tirup	1150–1350	29
Gråbrødrekloster	Ribe	1250–1536	15
Ribe Domkirke		900–1738	9
St. Jürgen	Lübeck	1270–1550	30
Gut Melaten	Aachen	1230–1550	56
Total	-	900–1812	352

*N* number of individuals with sufficient endogenous DNA

^†^ also known as St. Jørgen

### Determination of cases and controls under strict selection criteria

Leprosy was diagnosed in the skeletal remains of 86 individuals from the Gut Melaten and Skt. Jørgensgården cemeteries using a combination of genomic and osteological methods (Table 2; Additional file 2: Tab. S1). Metagenomic screening for the presence of *M. leprae* reads revealed 12 individuals from Gut Melaten and 50 individuals from the Skt. Jørgensgården cemetery to have been infected. *M. leprae* DNA was not detected at any other site (Additional file 2: Tab. S1). In addition to individuals positive for the pathogen, 24 individuals (17 from Gut Melaten and seven from Skt. Jørgensgården) were assigned to the case group based on the presence of skeletal lesions indicative of leprosy. The remaining 38 individuals from the leprosaria-associated cemeteries could not be confirmed as leprosy-affected and their case status was considered uncertain. Subsequently, they were removed from further analysis. Out of 228 individuals from the remaining cemeteries, 225 were assigned the control status based on the absence of genetic and osteological evidence of leprosy (Table 1). The disease status of two individuals from Tirup kirke and one individual from Horsens Ole Worm was considered uncertain and these individuals were excluded from the analysis.

**Table 2:** Number of leprosy cases detected with osteological and genetic methods

	Gut Melaten [N]	Skt. Jørgensgården [N]
Osteological evidence only	17	7
Genetic evidence only	1	25
Both methods	11	25
Total N	29	57

N - number of individuals

**Fig. 2:**
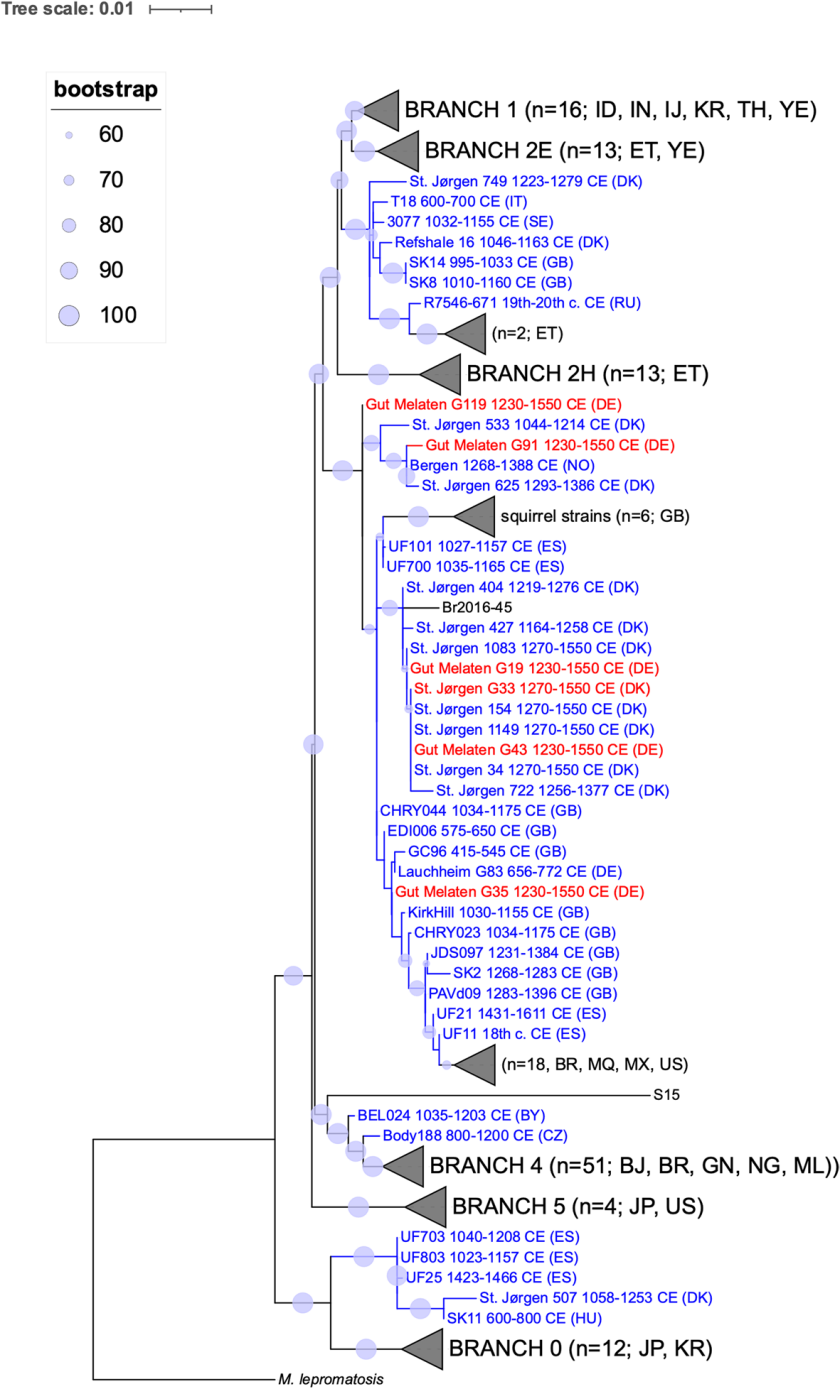
A maximum-likelihood tree illustrating the phylogenetic position of the *M. leprae* strains. The strains from Gut Melaten and Skt. Jørgensgården are shown in red. Previously published medieval strains are marked in blue and modern *M. leprae* are shown in black. Circles at each node represent bootstrap support over 500 replications. Only branches with bootstrap support of at least 60 are marked. The tree includes 183 strains (139 modern and 44 medieval) (Additional file 2: Tab. S2). *M. lepromatosis*, which is known to cause leprosy in America, was used as an outgroup. The tree was constructed using a set of 3124 previously published informative SNPs [15] (Additional file 3: Fig. S2). Country codes can be found in the Additional file 2: Tab. S3. Dates are provided for ancient strains. The tree was visualized with iTOL [42]

Furthermore, six partial *M. leprae* genomes (51–97%, 1.5–11.7x) were reconstructed, five from Gut Melaten and one from Skt. Jørgensgården (Table 3). The alignment of sequence reads to *M. leprae* genome demonstrated deamination signatures at terminal positions, supporting the authenticity of their ancient origin (Additional file 3: Fig. S1). In a phylogenetic tree, all new genomes clustered within the diversity of branch 3 (Fig. 2, Additional file 3: Fig. S2). The strains from Melaten fell on separate subbranches. As the genomes of G91 and G35 were only partially reconstructed (60% and 20% of the informative SNPs were covered), the placement of these strains within the phylogenetic tree should be interpreted with caution.

**Table 3:** Mapping statistics for six samples aligned to the *M. leprae* reference genome

Site	Grave ID	N of reads^#^	Mean coverage	Coverage 1x [%]	Coverage 2x [%]	Coverage 3x [%]
Gut Melaten	G19	152,884	4.22x	85.63	68.73	52.86
Gut Melaten	G119	111,253	3x	83.66	63.13	43.68
Gut Melaten	G43	376,821	11.71x	97.04	94.8	91.18
Gut Melaten	G91	103,455	2.38x	66.19	38.36	20.55
Gut Melaten	G35	68,098	1.47x	51.24	21.18	7.87
Skt. Jørgensgården	G33	232,312	5.03x	95.96	90.42	79.76

^#^The BAM files were filtered for a mapping quality of > 30

**Fig. 3:**
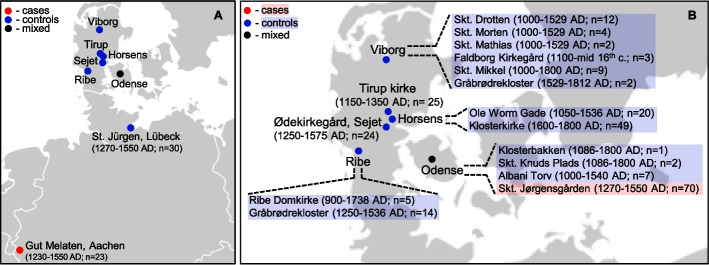
Map of archaeological sites from which skeletal remains included in the case–control study were excavated. Archaeological dates and the number of individuals are presented in brackets. The leprosy status of individuals at each site included in the association study is color-coded: red for cases and blue for controls. The locations of all sites are shown in panel A. Panel B presents the names and locations of all Danish sites

Subsequently, all individuals were evaluated for kinship to avoid introducing bias stemming from close relatedness. Kinship was detected for four pairs of individuals in the control group (Additional file 2: Tab. S4) and therefore, one individual from each pair (*N* = 4) was excluded from further analysis, resulting in 86 leprosy-affected cases and 221 controls (Additional file 2: Tab. S1). Of these 307 individuals, HLA profiles could be produced for 271 (62 cases and 209 controls) (Additional file 2: Tab. S1). Additionally, 31 case genotypes generated in a previous study [41] were incorporated which led to a final dataset of 93 cases and 209 controls (*N* = 302) for the association study (Table 4, Fig. 3 , and Additional file 2: Tab. S1).

**Table 4:** Number of individuals included in the case–control association analysis

Site	Location	N	Case–control status
Skt. Jørgensgården^†^	Odense	70^#^	cases
Skt. Knuds Plads		2	controls
Albani Torv		7	controls
Klosterbakken		1	controls
Skt. Trinitatis/Drotten	Viborg	12	controls
Skt. Morten		4	controls
Skt. Mathias		2	controls
Skt. Mikkel		9	controls
Gråbrødrekloster		2	controls
Faldborg Kirkegård		3	controls
Klosterkirke	Horsens	49	controls
Ole Worms Gade		20	controls
Ødekirkegård	Sejet	24	controls
Tirup kirke	Tirup	25	controls
Gråbrødrekloster	Ribe	14	controls
Ribe Domkirke		5	controls
St. Jürgen	Lübeck	30	controls
Gut Melaten	Aachen	23	cases
Total		302#	

*N* number of individuals included in the case–control study (93 cases and 209 controls)

^†^ also known as St. Jørgen

^#^ includes 31 individuals with HLA genotypes reported in a previous study by Pierini et al. [41]

### Genetic homogeneity within the medieval sample and temporal genetic continuity in Europeans

**Fig. 4:**
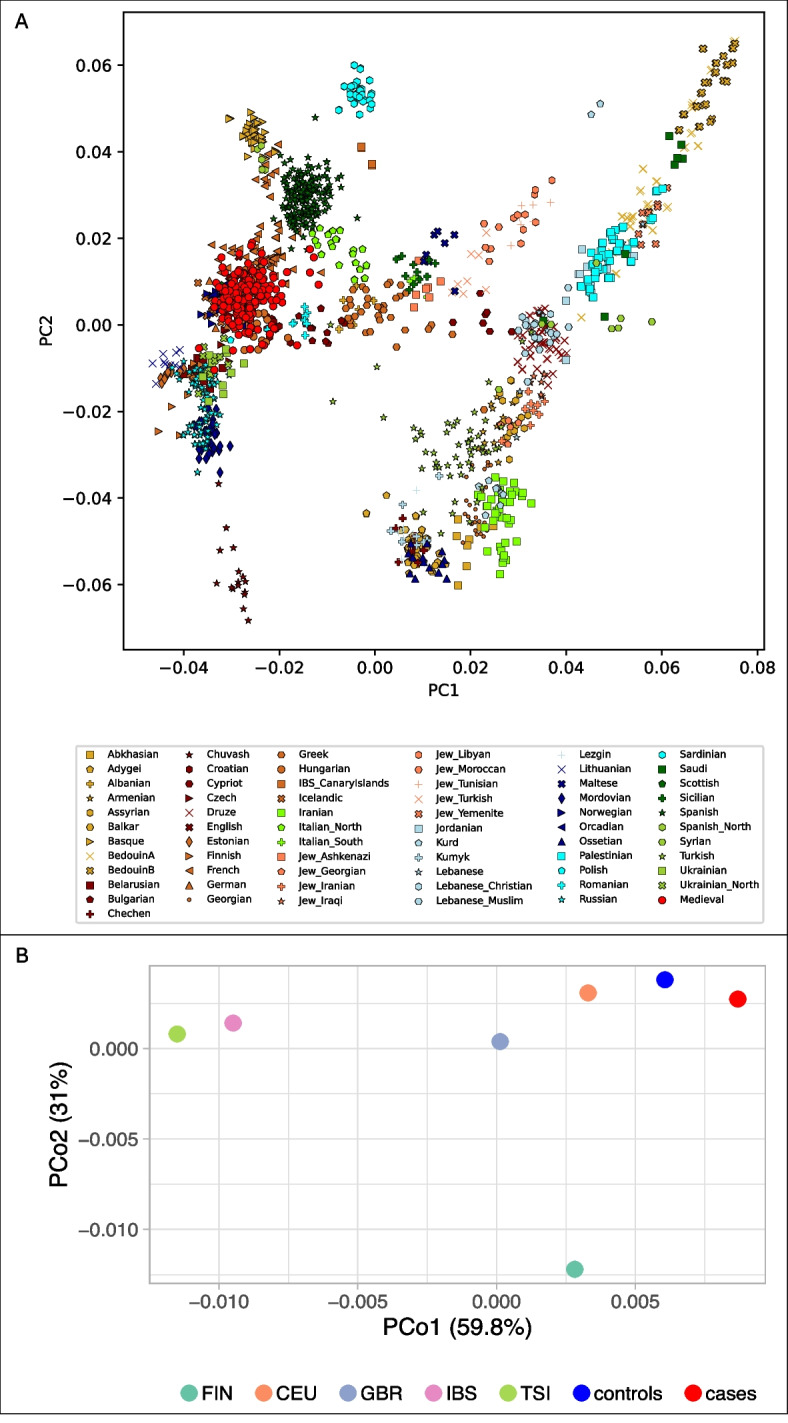
Analysis of differentiation between medieval and modern populations. **A** – PCA of 178 medieval individuals, based on SNPs from the 1240 k SNP panel, projected onto the first two principal components calculated from 66 present-day West-Eurasian populations. **B** – PCoA of 172 medieval individuals based on the pairwise FST values for HLA genotypes. Variance accounted for each principal coordinate is shown within axis labels

To evaluate potential stratification between cases and controls, principal component analysis (PCA) was performed based on a set of genome-wide SNPs. The PCA showed that all individuals grouped together within the diversity of western and northern Europeans (Fig. 4A, Additional file 3: Fig. S3). When cases and controls were considered separately, the groups overlapped on the PCA plot along both axes, including the individuals from the later dating Klosterkirke burials (Additional file 3: Fig. S4 and S5). Furthermore, there were no significant differences in HLA allele frequencies between the individuals from Klosterkirke and the remaining controls (Additional file 2: Tab. S5). These results indicate that there is no substantial genome-wide diversity between any of the groups and a comparison of allelic frequencies in a case–control association study is warranted. Furthermore, population differentiation was measured with the fixation index (FST) of the HLA alleles. The FST between medieval cases and controls was low (< 0.004), suggesting that the allele pools of the two groups were highly similar. All medieval individuals pooled together exhibited very low levels of differentiation from present-day northern and central Europeans (Fig. 4B), which points to population continuity since the Middle Ages.

### Association analysis – primary analysis

In comparison to research conducted in present-day populations, the sample size in this study is relatively small. Therefore, testing of all observed alleles would drastically reduce the power of detecting significant association signals (Additional file 2: Tab. S6). To overcome this limitation, a targeted approach was taken, i.e., 13 alleles previously linked with leprosy in present-day non-European populations were selected for the association analysis (primary analysis) (Additional file 2: Tab. S7). The alleles were chosen based on a strong association (odds ratio (OR) of at least 2 or less than 0.5) reported in studies with a robust sample size of at least 200 cases and 200 controls. As a large proportion of the HLA results have been reported at the first-field resolution, which groups alleles of highly similar sequence, the association analysis was performed at this level.

Across the 13 selected alleles, the frequency of HLA-B*38 differed significantly between cases (3.9%) and controls (0.3%) (Fisher’s exact test; *p* < 0.05). The signal diminished after correction (the *p*-value increased from 0.006 to 0.07 (Table 5) likely due to the insufficient statistical power achieved for this allele (post hoc: given α = 0.05, sample size and effect size, achieved power (1-β) < 0.6). Nevertheless, the association was confirmed using simulations where case–control labels were randomly permuted within the dataset (1000 permutations) (Additional file 3: Fig. S6). Fewer than 5% of permuted *p*-values matched or exceeded the observed *p*-value, indicating that the observed difference in B*38 allele frequency is unlikely to have occurred by chance. Furthermore, although not statistically significant, trends were observed for three alleles. HLA-B*44 and DRB1*15 were more frequent in cases in comparison to controls: 14.7% vs. 8.6% and 23.6% vs. 17.2%, respectively. HLA-C*08, on the other hand, appeared only in controls (1.7%). Statistical power analysis indicated that this study is underpowered (achieved power < 0.35) to reliably detect the observed frequency shifts for these alleles (Additional file 2: Tab. S8). Allele frequencies for the remaining 10 alleles were comparable between cases and controls.

**Table 5:** Results of the case–control association analysis between 13 selected HLA alleles and leprosy

HLA allele	CASES (*n* = 93)		CONTROLS (*n* = 209)		*p*-value	pc	OR	CI low	CI up
	N	Fr	N	Fr					
A*29	2	0.014	5	0.014	1.000	1.000	1.028	0.097	6.368
B*07	22	0.171	54	0.150	0.573	1.000	1.169	0.645	2.060
B*14	0	0.000	1	0.003	1.000	1.000	0.000	0.000	108.955
B*38	5	0.039	1	0.003	0.006^*^	**0.073**	14.425	1.592	685.839
B*44	19	0.147	31	0.086	**0.061**	0.736	1.836	0.940	3.511
B*49	0	0.000	2	0.006	1.000	1.000	0.000	0.000	14.927
C*02	5	0.036	17	0.048	0.808	1.000	0.747	0.211	2.165
C*05	10	0.073	23	0.065	0.841	1.000	1.126	0.465	2.544
C*08	0	0.000	6	0.017	**0.192**	1.000	0.000	0.000	2.176
C*15	5	0.036	12	0.034	1.000	1.000	1.073	0.290	3.351
DRB1*10	1	0.007	1	0.003	0.495	1.000	2.462	0.031	193.953
DRB1*15	33	0.236	59	0.172	**0.125**	1.000	1.489	0.889	2.465
DQB1*04	4	0.028	18	0.052	0.339	1.000	0.532	0.129	1.655

*N* number of alleles, *Fr* frequency, *pc p*-value corrected for multiple testing using Bonferroni correction (number of tests = 13), *CI low* confidence interval lower bound of OR, *CI up* confidence interval upper bound of OR, significant results and nonsignificant trends are highlighted in **bold**

*p-value statistically significant

### Association analysis – explorative analysis

To investigate the medieval HLA dataset for potentially novel associations, an explorative association study was performed for all the remaining alleles observed across the six classical HLA loci (Additional file 2: Tab. S9). Nominally significant differences in allele frequencies were noted for HLA-A*23, DRB1*04, and DRB1*13 (Table 6). Whilst A*23 and DRB1*04 were more frequent among cases (4.9% vs. 0.3% and 24.3% vs 15.7%), the frequency of DRB1*13 was over two times higher in controls relative to cases (16.9% vs. 7.1%). In addition to the nominally significant associations, nonsignificant trends were observed for one class I and three class II alleles (Table 6). Similar to the trends noted in the primary analysis, the achieved statistical power was low (< 0.6) (Additional file 2: Tab. S8).

**Table 6:** Exploratory case–control association analysis: nominally significant results and non-significant trends

HLA allele	CASES (*n* = 93)		CONTROLS (*n* = 209)		*p*-value	OR	CI low	CI up
	N	Fr	N	Fr				
A*23	7	0.049	1	0.003	0.001^*^	18.75	2.37	848.18
A*26	7	0.049	7	0.019	0.074	2.64	0.78	9.01
C*06	6	0.044	32	0.091	0.092	0.46	0.15	1.15
DRB1*03	12	0.086	51	0.148	0.074	0.54	0.25	1.07
DRB1*04	34	0.243	54	0.157	0.037^*^	1.72	1.03	2.86
DRB1*13	10	0.071	58	0.169	0.006^*^	0.38	0.17	0.78
DPB1*04	27	0.443	118	0.578	0.078	0.58	0.31	1.07
DPB1*452	2	0.033	0	0.000	0.052	Inf	0.63	Inf

*N* number of alleles, *Fr* frequency, ^*^p-value statistically significant, *CI low* confidence interval lower bound of OR, *CI up* confidence interval upper bound of OR, *Inf* infinity due to the frequency of 0 in one of the groups; significant results are highlighted in **bold**

In order to assess the potential confounding effect of DNA damage on HLA associations, two indicators of DNA preservation were compared between cases and controls: the λ parameter from damage modelling and the mean terminal deamination rate (average of 5′ and 3′ misincorporation rates) (Additional file 3: Fig. S7). While Mann–Whitney U tests indicated significant differences between groups (*p* = 8.04e-05 for λ; *p* = 0.016 for terminal damage), the distributions of both parameters showed substantial overlap and no clear evidence of systematic skewed or group-specific damage patterns. To control for the effect of damage-related confounding, a multivariate model was employed. Case/control status was defined as a binary outcome, with predictor variables including four HLA alleles of interest (B*38, A*23, DRB1*04, and DRB1*13), along with three measures of DNA preservation: the λ parameter, mean terminal deamination rate and the endogenous DNA content. The HLA alleles were selected based on prior analyses. In line with the previous observations, B*38, A*23, and DRB1*04 remained associated with the case status, while DRB1*13 was protective after accounting for DNA damage (Additional file 2: Tab. S10). The odds ratios derived from this model were consistent with those obtained in the unadjusted association analyses, thereby confirming that observed HLA effects were not driven by DNA damage.

### Heterozygosity and allelic richness in cases and controls

To further explore differences between cases and controls, heterozygosity and allelic richness were calculated at first-field resolution and compared between the groups (Table 7). Heterozygosity was calculated as the proportion of heterozygous individuals within each group (cases or controls). Allelic richness was calculated using the rarefaction method that accounts for the differences in sample sizes. While the allelic richness values were similar in both groups, heterozygosity was consistently lower in controls relative to cases for all class I and II alleles, except for the HLA-DRB1 locus. The difference, however, was statistically significant only for HLA-A.

**Table 7:** Diversity parameters for each HLA locus at the first-field resolution

Locus	Group	Sample size	Htzg	pc	N of alleles	Allelic Richness
A	control	176	0.76	0.9	17	13.8
	case	63	0.90		13	13
B	control	170	0.81	0.5	23	18.3
	case	56	0.91		21	21
C	control	167	0.73	1	16	12.8
	case	60	0.82		11	11
DRB1	control	165	0.87	1	14	11.8
	case	63	0.84		12	12
DQB1	control	166	0.69	1	5	5
	case	66	0.76		5	5
DPB1	control	88	0.53	1	19	8.3
	case	16	0.69		14	14

*Htzg* heterozygosity, *pc p*-value corrected for testing on six HLA loci

### Binding prediction of *M. leprae* proteins

**Fig. 5:**
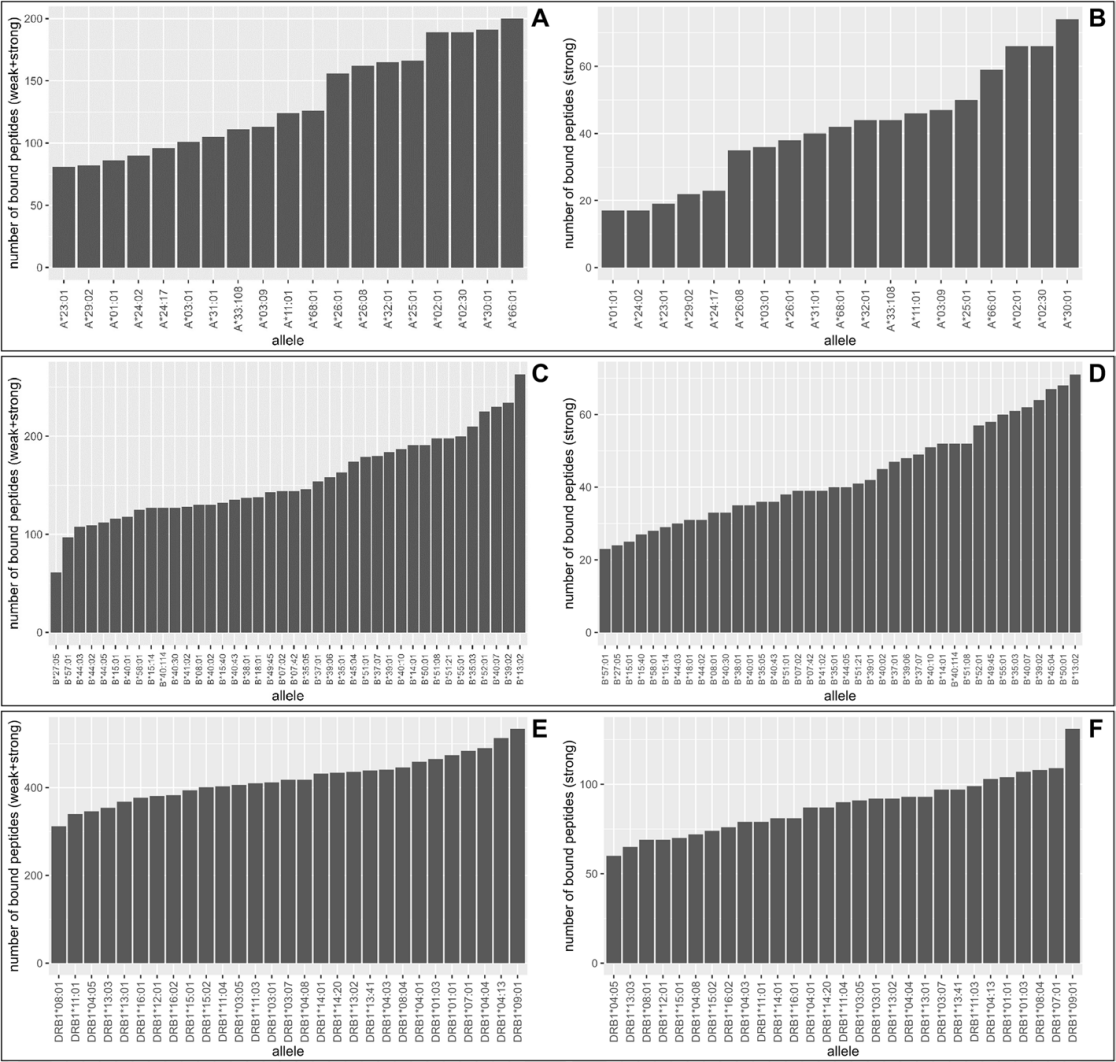
The number of peptides predicted to be bound by HLA-A (**A**-**B**), HLA-B (**C**-**D**), and HLA-DRB1 (**E**–**F**). Bound peptides were determined based on %RankEL scores either by using a strong binding threshold giving the strongly bound peptides or a weak binding threshold giving both strongly and weakly bound peptides

The presentation of specific peptides on the cell surface, which is the major function of HLA molecules, is commonly assumed to be the causal factor for HLA-mediated resistance or susceptibility to diseases [43, 44]. To investigate the peptide binding properties of the HLA alleles associated with leprosy in this study, a computational binding prediction was performed for all HLA alleles observed in the relevant loci (HLA-A, B, and DRB1) and 46 antigenic *M. leprae* proteins (Additional file 2: Tab. S11, Fig. 5).

Among the alleles that were observed at significantly different frequencies in cases and controls for both association analyses, HLA-A*23:01 (Fig. 5A and B), B*44 (Fig. 5C and D), and DRB1*15:01 (Fig. 5E and F) are placed on the lower end of the spectrum. While B*38:01 deviates from this trend, it still binds relatively few peptides, especially when considering strong binders (Fig. 5D). HLA-DRB1*04 alleles called at the second-field resolution in the medieval sample (53% of all alleles) were represented mostly by DRB1*04:01 and 04:04, which are also the most common DRB1*04 alleles in today’s Germans (Allele Frequency Net Database (AFND): Germany DMKS (*N* = 3,456,066)). In the binding prediction analysis, multiple DRB1*04 alleles were included, all of which were predicted to bind a considerably different number of peptides, with DRB1*04:01 and 04:04 in the middle of the spectrum.

The potentially protective HLA-DRB1*13 allele family is represented by two alleles, DRB1*13:01 and DRB1*13:02. Although they differ markedly in terms of the number of weakly bound peptides, both are placed above average when focusing on strongly bound peptides (Fig. 5F).

### Temporal shifts in HLA allele frequencies

Genetic continuity between medieval individuals and modern Germans (Fig. 4A and B, Additional file 3: Figures S3 and S4) allowed an investigation of potential shifts in HLA diversity over time. HLA allele frequencies were compared between the medieval controls and a large cohort of modern Germans (Allele Frequency Net Database (AFND): Germany DMKS (*N* = 3,456,066)) (Additional file 2: Tab. S12). The German population was used as the modern Danish cohort available in AFND included only 55 individuals [45]. Overall, the allele frequencies are similar between the two groups. The proportions of HLA-C*04, DRB1*11, DQB1*05, and DPB1*02 have increased since the Middle Ages. On the contrary, HLA-C*03 and DQB1*06 are less common in present-day Germans relative to medieval individuals (Table 8). The shift is particularly pronounced for HLA-C*03, with a difference of over 10%.

**Table 8:** HLA alleles which exhibit a temporal shift (> 5%) in frequency

	Frequency [%]	
HLA allele	Medieval controls	Modern Germans
C*03	25	14.24
C*04	6.25	11.5
DRB1*11	4.93	11.96
DQB1*05	12.07	17.12
DQB1*06	33.05	25.7
DPB1*02	8.33	14.52

## Discussion

In this study, HLA profiles of 302 individuals were produced and examined to uncover links between human genetic factors and susceptibility to leprosy in medieval Europe. It is one of very few disease association studies performed in past populations until now [17, 46]. In addition, six new partial *M. leprae* genomes were reconstructed and all clustered in branch 3, the most dominant *M. leprae* clade in medieval Europe [18].

### Primary analysis: HLA-B*38 involved in leprosy susceptibility in medieval Europeans

In the primary analysis of this study, 13 variants previously described to be associated with leprosy were tested (Table 5). Of these, HLA-B*38 was confirmed as a risk allele, which was reported for present-day Brazilians and Vietnamese [47, 48]. It is considered a susceptibility factor for several autoimmune conditions, such as psoriatic arthritis [49], pemphigus vulgaris [50, 51], and ankylosing spondylitis (AS) [52]. It is also thought to contribute to the development of various adverse reactions to certain medications [53–55]. The overall picture portrays the HLA-B*38 allele as a potentially detrimental allele.

### Primary analysis: frequency trends for HLA-B*44, DRB1*04, and DRB1*15

The frequency of HLA-B*44 was higher in cases (Table 5), showing a trend that was opposite to the report previously made for the Vietnamese population [48]. This discrepancy in the association direction (risk in Europeans vs. protection in Vietnamese) likely reflects differences at the second-field resolution (protein level), meaning that the disease susceptibility could be linked to different HLA-B*44 alleles, e.g., B*44:03 in the Vietnamese [48] and B*44:02 in medieval Europeans (B*44:02 was by far the most frequent B*44 allele in the medieval sample (*N* = 17)).

A strong trend was also noted for HLA-DRB1*15 (23.6% vs 17.2%). In the ancient dataset, DRB1*15:01 was the most common DRB1*15 allele at the two-field resolution (*N* = 47). Also in modern Europeans, DRB1*15:01 is present at a high frequency of 13.3% followed by DRB1*15:02 with 0.7% ((AFND): Germany DMKS (*N* = 3,456,066)) [45]. HLA-DRB1*15:01 is an allele that is most consistently associated with leprosy across various populations [17, 33–37]. This allele was also identified to significantly increase the likelihood of developing multiple sclerosis (MS) [56]. On the contrary, carriers of DRB1*15:01 displayed an increased protection against type I diabetes [57] and rheumatoid arthritis (RA) [58]. HLA-DRB1*15:01 was shown to be introduced into the European gene pool via admixture of the Steppe populations at the end of the Neolithic period [59], highlighting the role of ancient migrations in the interplay between host genetic factors and disease susceptibility. Although the previous association of this allele with leprosy risk for medieval Europeans [17] could not be confirmed in this study, this is likely due to the insufficient sample size (achieved power = 0.23) (Additional file 2: Tab. S8). Due to the strict allele calling, it is possible that homozygous calls are underrepresented, which would result in a lower allele frequency.

### Exploratory analysis: Identification of potentially novel leprosy-associated variants in Europeans

In the exploratory analysis, three potentially novel variants, HLA-A*23, DRB1*04, and DRB1*13, were detected as they showed nominally significant associations. HLA-DRB1*04 was substantially more frequent in leprosy-affected individuals (Table 6). This allele had been previously identified as a protective factor in present-day Argentinians, Brazilians, Vietnamese, Japanese (DRB1*04:01), and Taiwanese (DRB1*04:05) [34, 35, 60–62]. Notably, DRB1*04 was linked to other mycobacterial disease – tuberculosis, illustrating that distinct DRB1*04 alleles can exert opposing effects within the same population. For instance, in Brazilians, 04:11 increased susceptibility, whereas 04:07 was protective [63]. These findings highlight that the influence of DRB1*04 on mycobacterial disease susceptibility is likely allele-specific and underscore the importance of distinguishing subtypes when interpreting associations.

To the best of our knowledge, HLA-A*23 has not been mentioned as a risk factor for leprosy before. The allele is relatively rare in Europe today, which is also reflected in the medieval sample (Table 6). HLA-DRB1*13, which protects against leprosy in this study, is much more common and considered a generally advantageous variant against various infectious diseases [64–66] and autoimmune conditions [67]. Since all three association results were only nominally significant, confirmation of the findings in an independent and larger cohort of medieval Europeans is required.

Substantial differences in frequency between cases and controls were also noted for five other HLA alleles (Table 6). The differences were, however, insignificant likely due to the limited sample size and the achieved power (Additional file 2: Tab. S8). Among the alleles were HLA-C*06 and DPB1*04 that were more frequent in controls. These alleles were also previously associated with leprosy protection in present-day India and China as well as Brazil, respectively [30, 68, 69].

### High heterozygosity at the HLA-A locus among leprosy-affected individuals

Heterozygosity was higher in leprosy cases in comparison to controls for all loci except for HLA-DRB1, although no difference remained significant after multiple testing correction (Table 7). This observation stands somewhat in contrast to the hypothesis of population heterozygote advantage, which states that individuals who are heterozygous at particular loci might be less susceptible to infectious diseases, as they can recognize and present a wider repertoire of antigens [70–72]. Based on this hypothesis, we would expect heterozygosity to be higher in controls. This study, however, focuses on one specific disease (leprosy) and does not evaluate the general health status of individuals. Furthermore, it was shown that in some cases, for instance during an HIV infection, homozygosity at certain HLA loci is advantageous [73].

### Binding prediction of *M. leprae* proteins

The presentation of specific peptides on the cell surface is thought to influence HLA-mediated resistance or susceptibility to diseases. When the number of *M. leprae*-derived peptides bound by the HLA-A, B, and DRB1 alleles was analysed, it was noted that the (potential) risk alleles (A*23, B*38, B*44, DRB1*15) tend to bind fewer peptides (Fig. 5). This was particularly apparent for A*23 that is among the HLA-A variants exhibiting the smallest peptide repertoire (Fig. 5A and B). In contrast, the two common alleles (i.e., DRB1*13:01 and DRB1*13:02) for HLA-DRB1*13, which was nominally associated with leprosy protection, bind more than the average number of peptides (Fig. 5E). The number of bound *M. leprae* peptides could potentially affect the efficiency of antigen recognition and contribute to the association of the alleles and leprosy susceptibility/protection. However, it should be noted that the number of HLA-presented peptides is not the only determinant influencing the immune response. There are likely other factors involved in the recognition and reaction to *M. leprae* that we could not account for in this study, such as expression levels of antigenic peptides or immunodominance [74].

### Allele frequency differences between medieval and modern controls as an indicator of the selection character of infectious diseases since the Middle Ages

**Fig. 6:**
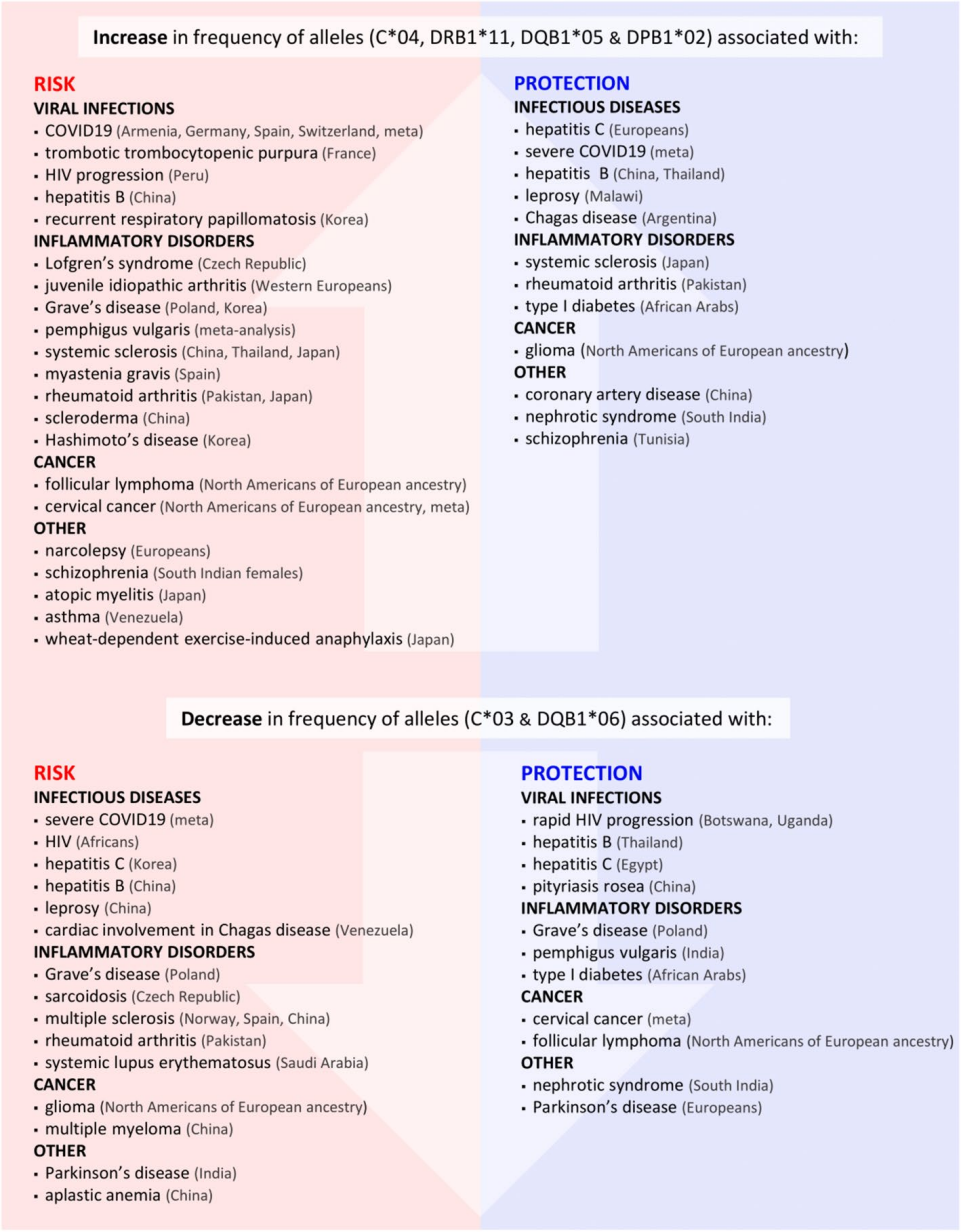
Six HLA alleles that exhibited a temporal shift in frequency are associated with increased risk to and protection against various diseases across different populations (Additional file 2: Tab. S13). meta – meta-analysis

Comparison of HLA allele frequencies between medieval controls and modern Germans showed similar values across most observed alleles (Additional file 2: Tab. S12), including those associated with leprosy in this study (Tables 5 and 6). This likely indicates genetic continuity through time and possibly balancing selection, which is well-documented for the HLA region [75]. It is, however, imperative to mention that the medieval control group includes individuals who lived and died in a period spanning several centuries. It is thus possible that selection varied at different time points [76], as epidemics of various infectious diseases swept across the continent. We noted substantial differences in frequencies (> 5%) of six alleles between medieval and modern populations (Table 8). All the alleles were previously shown to influence susceptibility to numerous diseases, including infections, autoimmunity, and inflammatory conditions (Fig. 6, Additional file 2: Tab. S13). The same alleles that are linked to infection susceptibility were also shown to play a role in inflammatory disorders. It was previously suggested that an evolutionary trade-off exists between infection resistance and autoimmunity/autoinflammation [77–79]. Whilst a large repertoire of recognized antigens provides a more efficient protection against infections, it also raises the risk of reacting to self-antigens [80]. In reality, the pattern of associations is highly complex and obscured by the intricacy of the pathophysiological mechanisms underlying different pathological conditions (Fig. 6 and the Additional file 2: Tab. S13).

### Addressing the limitations and potential of case–control association studies in past populations

The study is not without limitations, which must be considered when interpreting the results. First, as the sample cohort examined here is of relatively small size, variants of small effect size were likely undetected. Whilst stringent selection criteria were applied for cases, it is possible that early-stage infections were undetected in the control group and, therefore, statistical power could be potentially reduced. However, if this were the case in this study, it would make the association findings even more convincing. Second, errors in genotyping could have theoretically influenced the HLA frequencies. The highly degraded nature of aDNA makes the HLA typing challenging. The semi-automated TARGT pipeline is currently the most suitable method for this kind of data [41]. To limit genotyping errors, the approach for the manual calling was strict, i.e., the allele was not called unless there was 100% identity between the reference and the consensus of the aligned sequences. No assumptions were made when HLA typing. OptiType [81] was used to support or reject a call for class I alleles where the manual call was ambiguous. Furthermore, we showed that the associations were not driven by aDNA damage (Additional file 2: Tab. S10).

It is also important to note that the pathogen screening strategy applied here relied on detecting *M. leprae* DNA in skeletal samples, which primarily reflects systemic, blood-borne infection. Individuals who were only transient asymptomatic carriers, harbouring *M. leprae* in the nasal mucosa, would thus not have been discovered. Although osteological analysis enabled identification of individuals with advanced leprosy, the molecular approach cannot distinguish early or asymptomatic infections from clinically manifest disease in individuals without skeletal lesions. Consequently, the case group includes both severe pathological cases and systemic infections of uncertain stage. Therefore, any HLA alleles identified as enriched among infected individuals should be interpreted as influencing susceptibility to acquiring or sustaining systemic *M. leprae* infection, rather than the development of clinical pathology or progression to severe skeletal manifestations. This limitation may bias our results towards alleles affecting infection risk, whereas variants associated with disease severity or progression are less likely to be captured in this dataset.

Lastly, this study was mostly explorative. Whilst such analysis can detect potentially novel associations, as done here, confirmation of the findings in an independent ancient cohort from Europe is necessary.

## Conclusions

Nowadays, Europe is free of many infectious diseases which used to be common in the Middle Ages. Therefore, the genetic variation influencing these diseases in present-day Europeans cannot be investigated. However, the development of the aDNA technology allows for such analyses to be performed in past populations [17, 46, 82–84]. This case–control association study between HLA alleles and leprosy in medieval Europeans revealed a statistically significant link between HLA-B*38 and susceptibility to *M. leprae* infection. Furthermore, three novel variants that were possibly involved in leprosy risk (HLA-A*23 and DRB1*04) or protection (HLA-DRB1*13) were detected. Identifying leprosy-associated variants can improve our understanding concerning the contribution of specific HLA alleles to the disease and potentially allow us to harness this knowledge in a medical setting.

## Methods

### Material

The individuals examined in this study were excavated from 16 Danish and two German archaeological sites dating between the 10^th^ and 19^th^ century AD (Table 1). The cemeteries of Skt. Jørgensgården in Odense (Denmark) and Gut Melaten in Aachen (Germany) were associated with institutions that housed leprosy-affected individuals in the Middle Ages [38–40]. The remaining sites are considered ordinary cemeteries associated with parishes, monasteries, and friaries (except for a multiple burial at Albani Torv) (Additional file 1: Skeletal_collections-literature). In total, based on the amount of endogenous DNA, duplicate rates, and the evaluation of samples for contamination (see Methods), 352 individuals were included in the analyses (Additional file 2: Tab S1). The material comprised 530 samples (280 petrous bones, 237 teeth, and 13 postcranial elements).

HLA genotypes for individuals from Skt. Jørgensgården, Odense published previously were also incorporated in the study (Tab. S7 in Pierini et al. [41]).

### DNA extraction, library preparation, and sequencing

All DNA samples were extracted and processed in a dedicated aDNA facility at the University of Kiel following the guidelines on contamination control in ancient DNA [85–87], using previously published protocols for aDNA extraction [88] and half-UDG library preparation [89]. Shotgun sequencing was performed on the Illumina HiSeq 6000 (2 × 100) platform of the Institute of Clinical Molecular Biology (IKMB) in Kiel.

### Read preprocessing, mapping and evaluation for authenticity and contamination

Adapter sequences were removed and paired-end reads were merged with ClipAndMerge v1.7.7. [90]. Shotgun sequence data was mapped to the human genome (build hg19) using BWA v0.7.12 [91] with a reduced mapping stringency parameter “-n 0.01” to account for mismatches in aDNA. Additionally, seeding was disabled by using the parameter “-l 300”, forcing BWA to perform a more exhaustive alignment without relying on a seed. Duplicated reads were removed with DeDup v0.12.2 [90].

To confirm the ancient origin of the sequences, terminal damage of the reads (C to T substitutions) was assessed with DamageProfiler [92]. After the validation, the first two positions from the 5’ and 3’-ends of the reads were trimmed. Furthermore, X-chromosome and mitochondrial DNA contamination were assessed with ANGSD and Schmutzi, respectively [93, 94] (Additional file 2: Tab. S1).

### Pathogen screening and phylogenetic analysis of *M. leprae*

All clipped and merged metagenomic datasets were screened for the presence of *M. leprae* DNA with the Megan Alignment Tool 0.3.0 (MALT) [95] (SemiGlobal alignment mode, identity threshold = 90%), using a curated database containing 27,730 bacterial genomes available at the NCBI platform (24.01.2019). Output alignments were inspected visually in MEGAN 6 [96] to identify samples positive for *M. leprae* reads. Competitive mapping was also carried out against 13 *Mycobacterium* references and two non-*Mycobacterium* outgroup species (Additional file 2: Tab. S14) to identify and remove non-specific reads, ensuring that only sequences specific to *M. leprae* were retained for analysis. BWA v0.7.15 [91] was used to perform the alignment with parameters typical for aDNA (-n 0.01 and l −300). Additionally, a mapping quality filter of 30 and an identity filter of 99% were applied. Duplicate reads and sequences shorter than 50 bp were removed.

Reads that aligned specifically to the *M. leprae* reference after competitive mapping were manually inspected in Integrative Genomics Viewer (IGV) v2.17.1 [97]. Alignments were checked for even read distribution, absence of stacking, and lack of excessive SNPs or insertions. The identity of these reads was further confirmed with BLAST v2.15.0 [98], using a significance threshold of E-value lower than 1e-5. Only those samples that contained at least 5 *M. leprae* reads in competitive mapping were considered as positive for the pathogen’s DNA.

Samples positive for the pathogen were then subjected to targeted in-solution capture to obtain more data for analysis [99]. Biotinylated 70 bp RNA baits (myBaits® from Arbor Biosciences) were designed for *M. leprae* TN strain (NC_002677.1) using 3 × tiling. The baits were filtered for low sequence complexity and duplicates, resulting in a set of 135,953 baits. Two rounds of hybridization capture were performed, which is standard in aDNA studies [99], according to the manufacturer’s instructions. The enriched libraries were sequenced on the Illumina HiSeq 6000 (2 × 100).

Subsequently, the samples were mapped against the *M. leprae* TN reference genome (NC_002677) with Burrows-Wheeler Aligner (BWA) v0.7.12 [91] using the same parameters as described above (-n 0.01 -l 300). Duplicated reads were removed with DeDup v0.12.2 [90] and the BAM was filtered for a mapping quality of at least 30. Mapping statistics were obtained with Qualimap v2.2.1 [100] using bamqc option. *M. leprae* strains with at least 50% of the genome covered with 1 × were included in subsequent phylogenetic analysis. The ancient origin of these strains was confirmed by evaluation of terminal read damage with DamageProfiler [92]. UnifiedGenotyper module of the Genome Analysis Toolkit (GATK) v3.6 [101] was used to generate a VCF file. A set of 3124 phylogenetically informative SNPs [15] was extracted from the VCF (min. coverage 3x, min quality 30, majority call 90%). The phylogeny was reconstructed with the maximum likelihood algorithm using RAxML [102] and a GTR-GAMMA mod. Bootstrapping of 500 replicates was applied. A total of 44 medieval and 139 modern strains was included in the analysis (Additional file 2: Tab. S2). *M. lepromatosis* was used as an outgroup.

### Determination of the “case vs. control” status

Determination of the disease status was performed based on palaeopathological analysis of skeletal remains and screening of metagenomic sequences for *M. leprae* DNA. The association of Gut Melaten and Skt. Jørgensgården cemeteries with medieval leprosaria was also considered (Additional file 3: Fig. S8).

Based on the work of Møller-Christensen and his successors [e.g., 5,12,38–40], it has been widely accepted that a combination of characteristic facial skeleton deformities (*facies leprosa*, rhinomaxillary syndrome) together with lesions of the extremities (atrophy of phalanges in hands and feet, periostitis of tibia and/or fibula) is considered pathognomonic of severe leprosy (lepromatous leprosy, LL). Therefore, all skeletal remains were assessed for the presence of such lesions. Osteological analysis of skeletons from Gut Melaten, St Jürgen and, partially, Skt. Jørgensgården were performed as part of previous studies [17, 40, 103].

For the leprosaria-associated cemeteries (Skt. Jørgensgården and Gut Melaten), individuals who exhibited skeletal lesions pathognomonic of leprosy and/or molecular evidence of infection were treated as “cases”. As both leprosaria were eventually transformed into either a general infirmary (Gut Melaten) [40, 104] or a cemetery for the poor (Skt. Jørgensgården) [105], it is possible that leprosy-unaffected individuals were among the people buried at the associated cemeteries. Therefore, the disease status for the individuals who showed neither molecular nor osteological signs of leprosy was considered uncertain, and they were excluded from further analysis.

Individuals from the remaining cemeteries were assigned the “control” status based on the absence of molecular and/or osteological evidence of leprosy. Two individuals from Tirup Kirke, who showed unspecific skeletal lesions suggestive of a systemic infectious disease, were excluded from the analysis due to the uncertain disease status.

### Population genetic analysis

Pseudo-haploid genotypes were generated at positions from the 1240 K and Human Origins (HO) SNP panels, commonly used for population genetic analyses, with SequenceTools 1.2.2 [106–108]. Here, an intermediate threshold of 20,000 SNPs was set and samples with at least 20,000 typed SNPs (from the 1240 K panel) were included in the principal component analysis (PCA). In total, genotype data for 178 medieval individuals was merged with a dataset of previously published 1138 individuals from 66 contemporary West Eurasian populations (in the Allen Ancient DNA Resource (AADR) dataset v50.0.p1) [109] using *mergeit* from the EIGENSOFT package. The PCA was performed with *smartpca* and the ‘lsqproject’ option [110].

### Kinship analysis

Kinship analysis was performed using READ [111] which infers kinship based on the proportion of non-matching autosomal genotypes. A minimum of 2500 SNPs is required to produce reliable results [111]. Therefore, individuals were examined for relatedness only in instances when the number of SNPs used for the calculations was > 2500.

### HLA targeted capture and typing

One sample per individual was subjected to targeted enrichment of the HLA exonic regions using a bait panel designed previously [112]. A sample was a good candidate for capture when the endogenous DNA content in the shotgun data exceeded one percent and the proportion of duplicated reads mapping to the human genome (hg19) was below 40 percent.

Individuals for which the HLA capture was successful were then typed with the semi-automated HLA-typing pipeline TARGT (Targeted Analysis of sequencing Reads for GenoTyping), designed for the analysis of low-coverage sequences typical of aDNA [41]. Six classical HLA loci were typed: class I (HLA-A, B, and C) as well as class II (HLA-DRB1, DQB1, and DPB1). Class I HLA alleles were also called using the OptiType software (available only for class I HLA alleles) [81] with default parameters. Results obtained with OptiType were used in instances where the call made with TARGT was ambiguous (two possible options). In such cases, the allele supported by Optitype was chosen. When OptiType did not support either of the two TARGT options, the allele was not called.

### HLA allele frequency calculation and statistical analysis

In addition to individuals that were successfully typed in this study, HLA calls for 54 individuals from Odense reported in a previous publication were included (Tab. S7 in Pierini et al. [41]). The sample sets from this and the previous analysis overlapped, so that HLA calls were available for 23 samples in both studies. The alleles were called using the same typing pipeline TARGT. They were, however, based on sequences from independent DNA extracts. For the overlapping individuals (*N* = 23), the calls were merged in a way that would increase the resolution of the calls. In instances of discrepancy, the allele supported by OptiType results generated in this study was preferred (class I HLA). For class II alleles, the call of a higher resolution was selected. Moreover, if a heterozygous call was made for a locus in one dataset while the other dataset suggested a homozygote, the heterozygous call was chosen. In such a scenario, it is likely that the homozygous call had resulted from limited data. The remaining 31 individuals successfully genotyped only in Pierini et al., 2020 were also included in the analysis.

Association analysis was performed with the Fisher’s exact test (two-tailed) using R v. 4.3.1 [113]. Correction for multiple testing was done in R v. 4.3.1 using the *mt.rawp2adjp* function from the *multtest* package [114]. Statistical power was calculated with G*Power v3.1.9.4 [115]. A permutation test was performed for alleles that showed a significant difference between cases and controls by randomly shuffling case–control labels 1000 times. For each permutation, the *p*-value of Fisher's exact test was calculated to generate a null distribution of *p*-values. The frequency difference between cases and controls was considered significant if the observed *p*-value fell within the lowest 5% of the null distribution.

To assess the potential confounding effects of DNA damage on HLA association results, we applied a multivariate model incorporating three metrics of DNA preservation: endogenous DNA content, the λ parameter from damage modelling, and the mean terminal deamination rate (calculated as the average of 5′ and 3′ misincorporation rates). The damage parameters (λ and deamination rates) were estimated using mapDamage 2.0 [116], based on half-UDG treated, HLA-captured libraries that were used for HLA genotyping. To evaluate whether these preservation metrics influenced HLA associations, we performed Firth’s logistic regression using the R package *logistf* [117]. The binary outcome variable was disease status (case/control), and predictor variables included four HLA alleles (B*38, A*23, DRB1*13, and DRB1*04), along with the three preservation covariates. Three separate models were carried out, each including one of the three HLA alleles to assess each allele’s effect independently.

### FST calculation

HLA genotype data of five European populations from the 1000 Genomes Project were incorporated in the analysis of genetic differentiation between medieval cases and controls as well as between the medieval sample and the present-day populations [118]. The modern populations used were Utah residents (CEPH) with Northern and Western European ancestry (CEU), the Iberian population from Spain (IBS), the Toscani from Italy (TSI), populations of Finland (FIN) as well as England and Scotland (GBR). Pairwise Weir-Cockerham FST estimations based on individuals with complete HLA-A, B, C, DRB1, and DQB1 genotypes at the first-field resolution were obtained with the *adegenet* and *hierfstat* packages in R v. 4.3.1 [119]. Pairwise FST values were used as a distance matrix for the calculations of the principal coordinates analysis (PCoA).

### Estimation of heterozygosity and allelic richness

Heterozygosity and allelic richness (i.e., rarefied allele counts) were calculated separately for cases and controls using the *hierfstat* package in R v. 4.3.1 [119]. Prior to any calculations, individuals with missing genotypes were removed. The analysis was performed for all six HLA loci (A, B, C, DRB1, DQB1, and DPB1). Differences in heterozygosity between cases and controls were evaluated with Fisher’s exact test (two-tailed) using R v. 4.3.1 [113].

### Computational binding prediction of *M. leprae* proteins

Computational prediction of peptide binding was performed for all HLA alleles observed at loci that show association with leprosy in this study (HLA-A, B, and DRB1) and *M. leprae* proteins that exhibit antigenic potential (Additional file 2: Tab. S11). For the HLA class I loci, HLA-A and B, peptide binding predictions were generated with NetMHCIIpan (4.1). NetMHCIIpan (4.0) was used for the class II locus (HLA-DRB1) [120]. The number of bound 9-mer peptides for class I HLA alleles and 15-mer peptides for class II alleles were calculated based on the %RankEL scores. Default thresholds of strong and weak binding were used for HLA class I (0.5% and 2%, respectively) and HLA class II (2% and 10%, respectively).

## Supplementary Information

Additional file 1. Skeletal collections – literature.

Additional file 2. Tables S1-S14.

Additional file 3. Figures S1-S8.

Additional file 4. Review history.

## Data Availability

Analysed sequences are available through the European Nucleotide Archive under Accession Number PRJEB66169: https://www.ebi.ac.uk/ena/browser/view/PRJEB66169 [121]. No custom code, beyond the scripts and packages mentioned in the Methods, was used in this manuscript.
