# Supplementary material for: Ancient DNA study provides clues to leprosy susceptibility in medieval Europe
Source: Genome Biol. 2026 Jan 16;27:4. doi: 10.1186/s13059-025-03925-8 (PMC12838506; doi:10.1186/s13059-025-03925-8)
Supplement: Supplementary file 1 — Additional file 1. Skeletal collections – literature. [file 13059_2025_3925_MOESM1_ESM.pdf]

Ancient DNA study provides clues to leprosy susceptibility in medieval Europe

Skeletal Collections – Literature

List of skeletal collections analysed in this study.

Site	Location	Dating (year AD)	References
Skt. Jørgensgården	Odense	1270-1550	Arentoft, 1999; Boldsen and Mollerup 2006; Danmarks Kirker 2001b
Skt. Knuds Plads		1086-1800	Christensen & Krogh, 1998; 2001; Boldsen, 1999; Christensen, 1988
Albani Torv		1000-1540	Arentoft, 1984; Bjerregaard, 2016; Danmarks Kirker 2001a; Haase, 2022; Olsen & Tarp, 2018; Tarp & Pedersen, 2018
Klosterbakken		1086-1800	Christensen & Krogh, 2001; Windmüller & Boldsen, 2003
Skt. Trinitatis/Drotten	Viborg	1000-1529	Keyes et al., 2007; Pedersen, 1993; Tarp, 2010a
Skt. Morten		1000-1529	Larsen, 2010; Tarp, 2011
Skt. Mathias		1000-1529	Hjermind 1999; 2001; 2012; Larsen, 2011; Mollerup, 1994
Skt. Mikkel		1000-1800	Boldsen 1979; Larsen 2022; Vellev 1979
Gråbrødrekloster		1529-1812	Hjermind & Larsen, 2020; Larsen & Hjermind, 2013
Faldborg Kirkegård		1100-mid 16th c.	Rasmussen, 2017
Klosterkirke	Horsens	1600-1800	Petersen, 2007; Tarp, 2010b; Vestergård, 2012
Ole Worms Gade		1050-1536	Danmarks Kirker, 2005; Klemensen, 2009; Pedersen, 2010
Ødekirkegård	Sejet	1250-1575	Danmarks Kirker, 2010; Kjærgård, 2006; Pedersen, 2008
Tirup kirke	Tirup	1150-1350	Danmarks Kirker, 2013; Kieffer-Olsen 1984
Gråbrødrekloster	Ribe	1250-1536	Andersen 2003; Jantzen et al. 1995
Ribe Domkirke		900-1738	Madsen, 2009, 2012; Søvsø, 2010;2020
St. Jürgen	Lübeck	1270-1550	Rieger et al., 2022
Gut Melaten	Aachen	1230-1550	Prescher & Wagner, 2016

REFERENCES

Andersen, L. (2003). Ribe Gråbrødrekloster – det sidste kapitel. Mark og Montre, 23-40.

Arentoft, E. (1984). Albani Torv, Odense, 1983. Beretning (Unpublished).

Arentoft, E. (1999). De spedalskes hospital: Udgravning af Sankt Jørgensgården i Odense. Odense Bys Museer.

Bjerregaard, M. M. (2016). OBM3183 Albani Torv, Odense sogn, Odense herred, tidl. Odense amt. Sted nr. 08.04.07. Sb.nr. 318. Kampagne: 26-05-2015. Available at: https://www.kulturarv.dk/fundogfortidsminder/Lokalitet/218584/

Boldsen, J.L. (1999). Beretning om Skeletfundene i forbindelse med OBM 9784 og OBM 9785. Unpublished Anthropological report, ADBOU, Syddansk Universitet.

Boldsen, J. L. (1979). Liv og død i middelalderens Viborg. Resultater fra skeletudgravningen på Sct. Mikkels Kirkegård. *MIV* 8, 76-85.

Boldsen, J. L., & Mollerup, L. (2006). Outside St. Jørgen: Leprosy in the medieval Danish city of Odense. *American Journal of Physical Anthropology, 130(3),* 344-351. https://doi.org/10.1002/ajpa.20363

Christensen, A. S. (1988). *Middelalderbyen Odense*. Aarhus University Press.

Christensen, J. T., & Krogh, M. G. (2001). OBM9397 Klosterbakken, Odense Sogn, Odense herred, Fyns amt, tidl. OPdense amt. Sted nr. 08.04.07. Sb.nr. 109. Unpublished Excavation report, Odense Bys Museer.

Christensen, J. T. & Krogh, M. G. (1998). OBM 9784 Skt. Knuds Plads I, Odense sogn, Odense herred, Fyns tidl. Odense amt. Stednr. 080407. Sb. nr. 100 (RAS O). (Unpublished).

Danmarks Kirker, (2001a). S. Albani Kirke. Odense Amt IX, bind 3, 1729-1749.

Danmarks kirker, (2001b). S. Jørgensgårdens Kirke. Odense Amt IX, bind 3, 1877-1884.

Danmarks Kirker, (2005). Vor Frue Kirke, XVI, bind 10, 6137-6144.

Danmarks Kirker, (2010). Sejet Kirke, XVII, bind 12, 1087-1094.

Danmarks Kirker, (2013). Tirup Kirke, XVII, bind 16, 1589-1596.

Haase, K. (2022). Albani Kirke og Kirkegård. En oversigt over de arkæologiske kilders udsagn til belysning af Skt. Albani Kirke og Kirkegårds historie i perioden ca. 900 til 1250. CENTRUM. Forskningscenter for centralitet. Rapport nr. 12 2022, Odense Bys Museer.

Hjermind, J. (2001). Sankt Mathias Kirke på Hjultorvet. *Viborg Bogen* 2001, 48-54.

Hjermind, J. (2012). VSM 09793: Hjultorvet, Sct. Mathias Kirkegård Syd. Unpublished excavation report. Viborg Museum.

Hjermind, J. (1999). VSM F906: ”Byens Træ”, Hjultorvet. Unpublished excavation report. Viborg Museum.

Hjermind, J., & Larsen, L. A. (2020). Viborg Gråbrødre Kloster. Det centrale klosteranlæg: Arkæologiske undersøgelser 2004 og 2008-12 *hikuin* 41 (eds. Hans Krongaard Kristensen, Morten Larsen & Jens Vellev), 75-106.

Jantzen, C., Kieffer-Olsen, J., Madsen, P.K., (1995). De små brødre. SKALK 2, 11-16.

Keyes, G.M. (2007). Middelalderlige begravelser til Sct. Drottens Kirke. Bygherrerapport 88. Viborg Museum.

Keyes, G. M., Iversen, M. G. & Hjermind, J. (2007): VSM 09264: Sct. Trinitatis/Drotten, Sct. Leonis Gade 12, Viborg. Unpublished excavation report. Viborg Museum.

Kieffer-Olsen, J. (1984). VKH 1201 Tirup Ødekirke. Unpublished excavation report.

Kjærgaard, A. (2006). Udgravningsrapport, Sejet Ødekirkegård. Unpublished excavation report. Horsens Museum.

Klemensen, M.F. (2009). HOM 1649 Ole Worms Gade og Havneallé. Unpublished excavation report. Horsens Museum.

Kristensen, H. Krongaard, (1987). Middelalderbyen Viborg. Projekt Middelalderbyen bind 4. Centrum.

Larsen, L. A. (2010). VSM 09715: Sct. Mortens Kirkegård. Unpublished excavation report. Viborg Museum.

Larsen, L. A. (2011). VSM 09793: Hjultorvet, Sct. Mathias Kirkegård Syd. Upubliceret forundersøgelsesrapport. Viborg Museum.

Larsen, L. A. (2022). VSM 10777: Nyt psykiatrihospital (Sct. Mikkel kirke og kirkegård). Unpublished trial excavation report. Viborg Museum.

Larsen, L. A., & Hjermind, J. (2013). VSM 09543 Viborg Gråbrødrekloster, Viborg sogn, Nørlyng herred, Viborg amt. Unpublished excavation report. Viborg Museum.

Madsen, H. A. (2009). ASR 13, Lindegården. Unpublished Anthropological report, ADBOU, Syddansk Universitet.

Madsen, H. A. (2012). ASR 13 II, Lindegården. Antropologisk beretning. Unpublished Anthropological report, Sydvestjyske Museer.

Mollerup, L. (1994). Beretning om de Middelalderlige skeletfund fra St. Mathias kirkegård, Viborg, VSM 804 E. Unpublished Anthropological report, Viborg Museum.

Olsen, T. B., & Tarp, P. (2018). OBM 3183, Albani Torv. Unpublished Anthropological report, ADBOU, Syddansk Universitet.

Petersen, C. G. (2007). Bygherrerapport II, HOM 1272, Klosterkirken. Unpublished Excavation report, Horsens Museum.

Pedersen, D. D. (2008). HOM 1046, Sejet Ødekirkegård. Unpublished Anthropological report, ADBOU, Syddansk Universitet.

Pedersen, D. D. (2010). HOM 1649, Ole Worms Gade. Unpublished Anthropological report, ADBOU, Syddansk Universitet.

Pedersen, V. J. (1999). VSM F902: Sct. Trinitatis/Drotten, Sct. Leonis Gade 10, Viborg. Unpublished excavation report. Viborg Museum

Rasmussen, M. V. (2017). VSM F029 Faldborg Kirke, Vindum sogn, Middelsom herred, Viborg amt. Unpublished Excavation report, Viborg Museum.

Søvsø, M. (2010). Tidligkristne begravelser ved Ribe Domkirke – Ansgars Kirkegård? Arkæologi i Slesvig | Archäologie in Schleswig, Museum Sønderjylland, Arkæologi Haderslev, Kiel, 13, 147-164.

Søvsø, M. (2020). Ribes klostre. *Hikuin*, *41*(41), 29–46.

Tarp, P. (2010a). VSM 09.264, Viborg Skt. Drotten. Unpublished Anthropological report, ADBOU, Syddansk Universitet.

Tarp, P. (2010b). HOM 1272, Horsens Klosterkirke. Unpublished Anthropological report, ADBOU, Syddansk Universitet.

Tarp, P. (2011). VSM 09.715, Viborg Skt. Morten. Unpublished Anthropological report, ADBOU, Syddansk Universitet.

Tarp, P., & Pedersen, D. D. (2018). OBM9776, Thomas B. Thriges Gade. Unpublished Anthropological report, ADBOU, Syddansk Universitet.

Vellev, J. (1979). Sct. Mikkels kirke i Viborg. MIV 8, 58-73.

Vestergård, F. (2012). Arkæologisk Udgravning, Beretning HOM 1272, Klosterkirken. Unpublished Excavation report, Horsens Museum.

Windmüller, E., & Boldsen, J. L. (2003). Beretning om skeletfund fra udgravningen OBM9397 Klosterbakken, Odense sogn, Odense herred, Fyns amt, tidl. Odense amt. Sted nr. 08.04.07. Sb.nr. 109. Unpublished Anthropological report. ADBOU, Syddansk Universitet
