# Supplementary material for: Ancient DNA study provides clues to leprosy susceptibility in medieval Europe
Source: Genome Biol. 2026 Jan 16;27:4. doi: 10.1186/s13059-025-03925-8 (PMC12838506; doi:10.1186/s13059-025-03925-8)
Supplement: Supplementary file 3 — Additional file 3. Figures S1-S8. [file 13059_2025_3925_MOESM3_ESM.pdf]

Title: Ancient DNA study provides clues to leprosy susceptibility in medieval Europe

Additional file 3

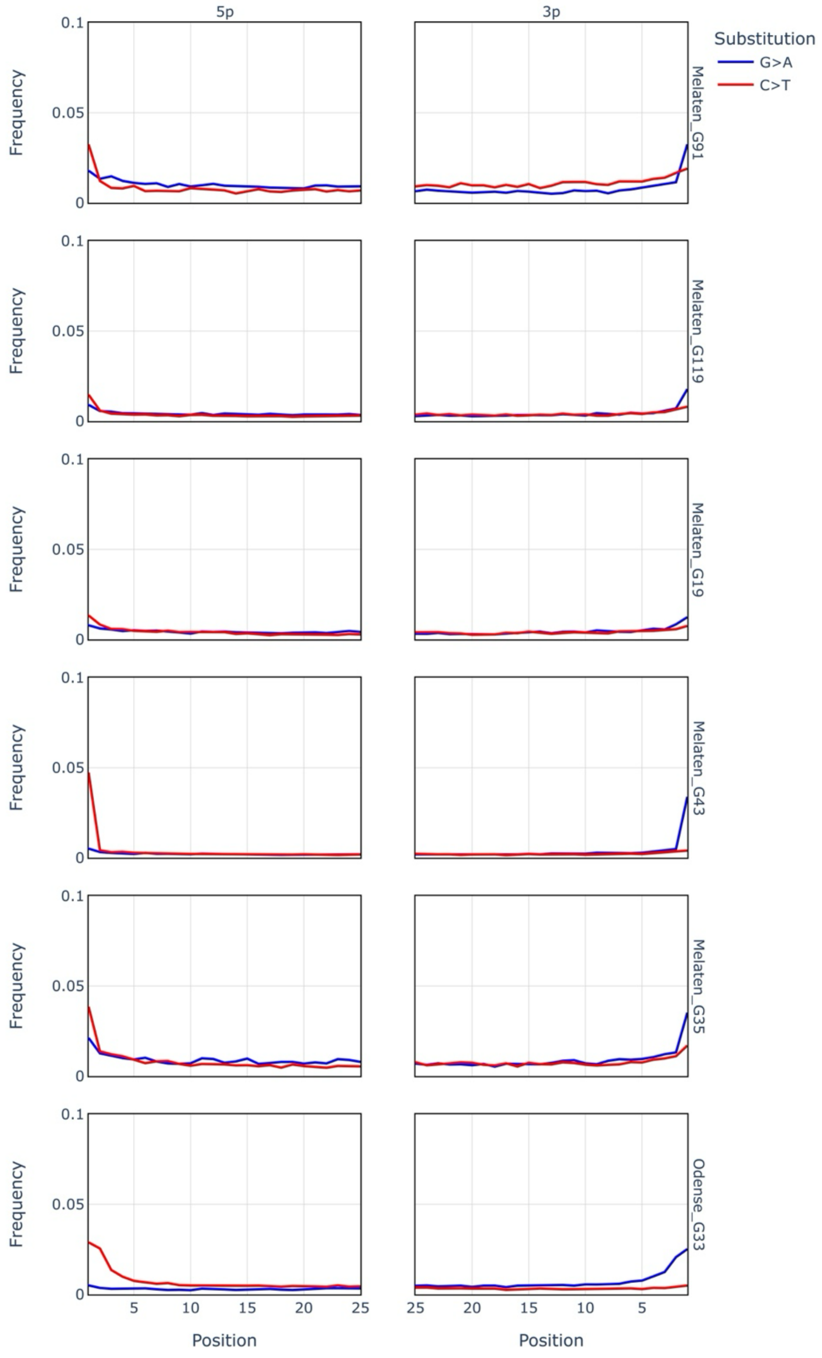

Figure S1. Comparison of nucleotide substitution rates across six samples from which partial *M. leprae* genomes were reconstructed. Sequences were aligned against *M. leprae* TN reference genome (see Methods).

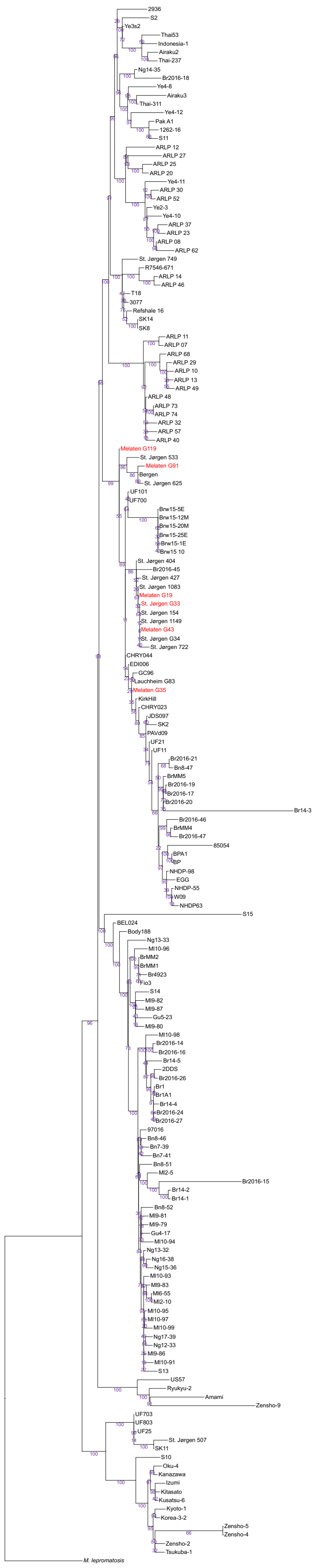

Figure S2. A maximum-likelihood tree illustrating the phylogenetic position of the M. leprae strains from Gut Melaten and St. Jørgen (shown in red). Purple numeric values at each node represent bootstrap support over 500 replications. The tree includes 183 strains (139 modern and 44 medieval) (Additional file 3: Tab. S2). *M. lepromatosis* was used as an outgroup.

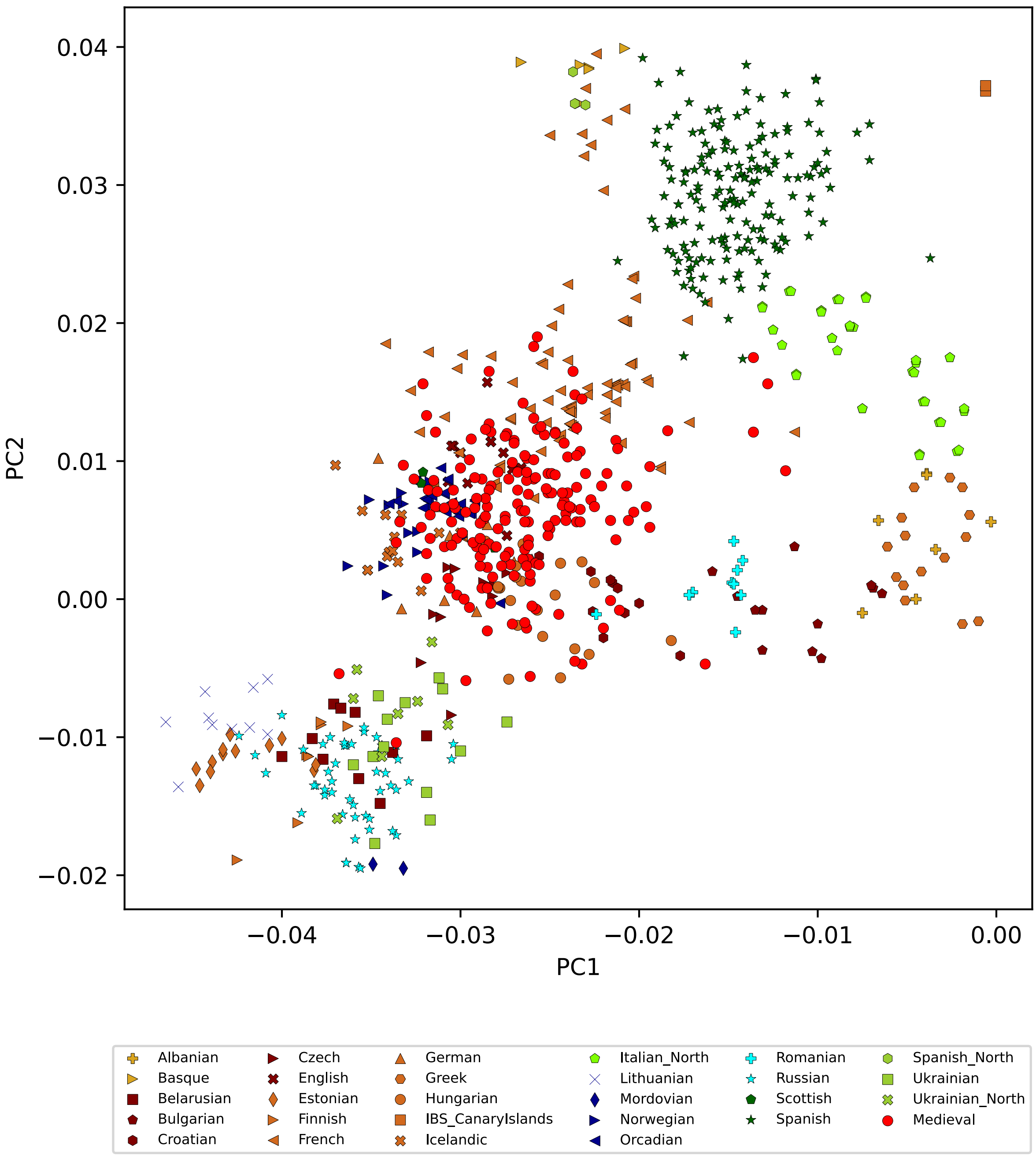

Figure S3. PCA of 178 medieval individuals, based on SNPs from the 2140k SNP panel, projected onto the first two principal components calculated from 66 present-day West-Eurasian populations. For clarity, only the closely-clustering 29 populations were plotted.

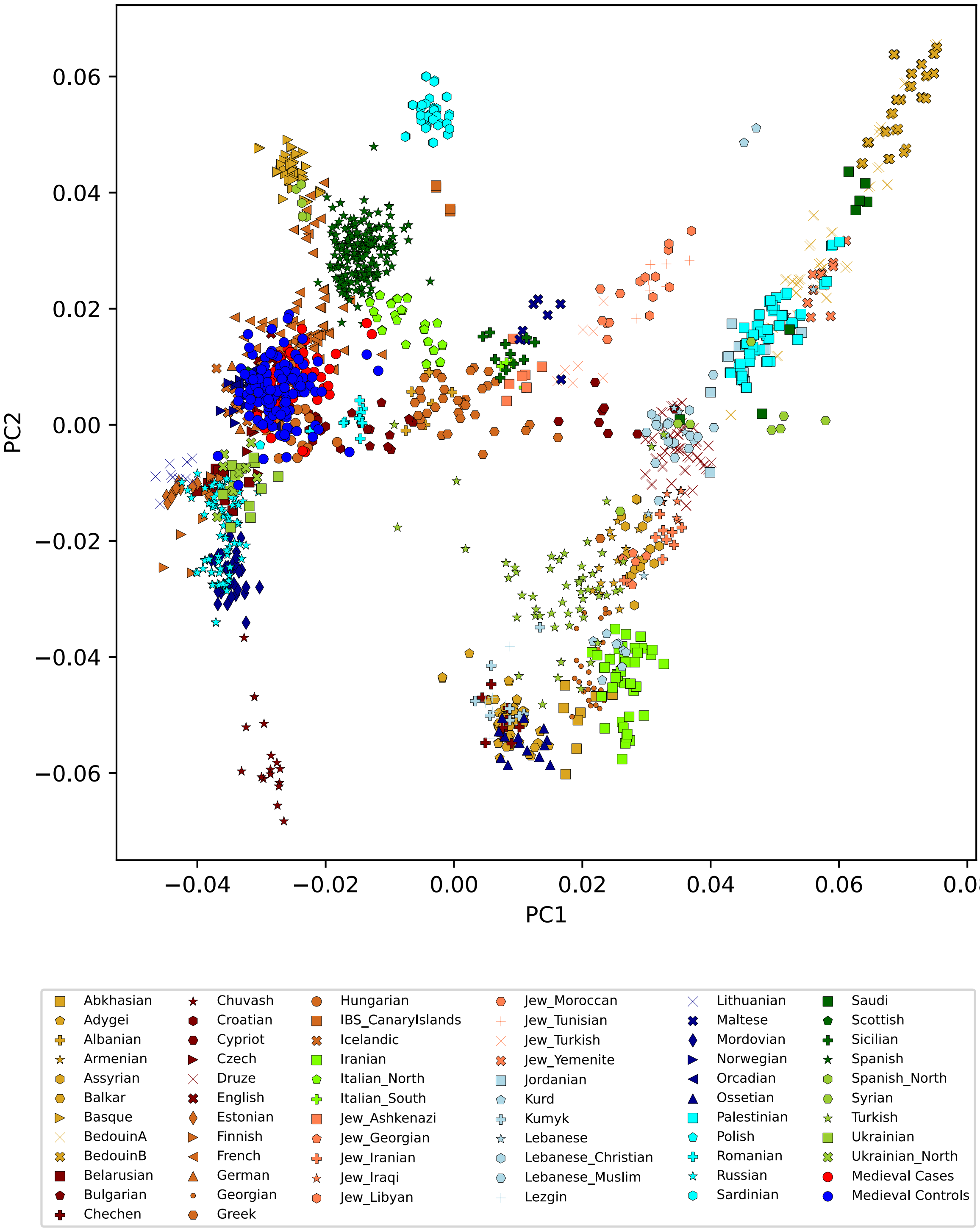

Figure S4. PCA of 58 cases (red circles) and 120 controls (blue circles) were projected onto the first two principal components calculated from 66 present-day West-Eurasian populations.

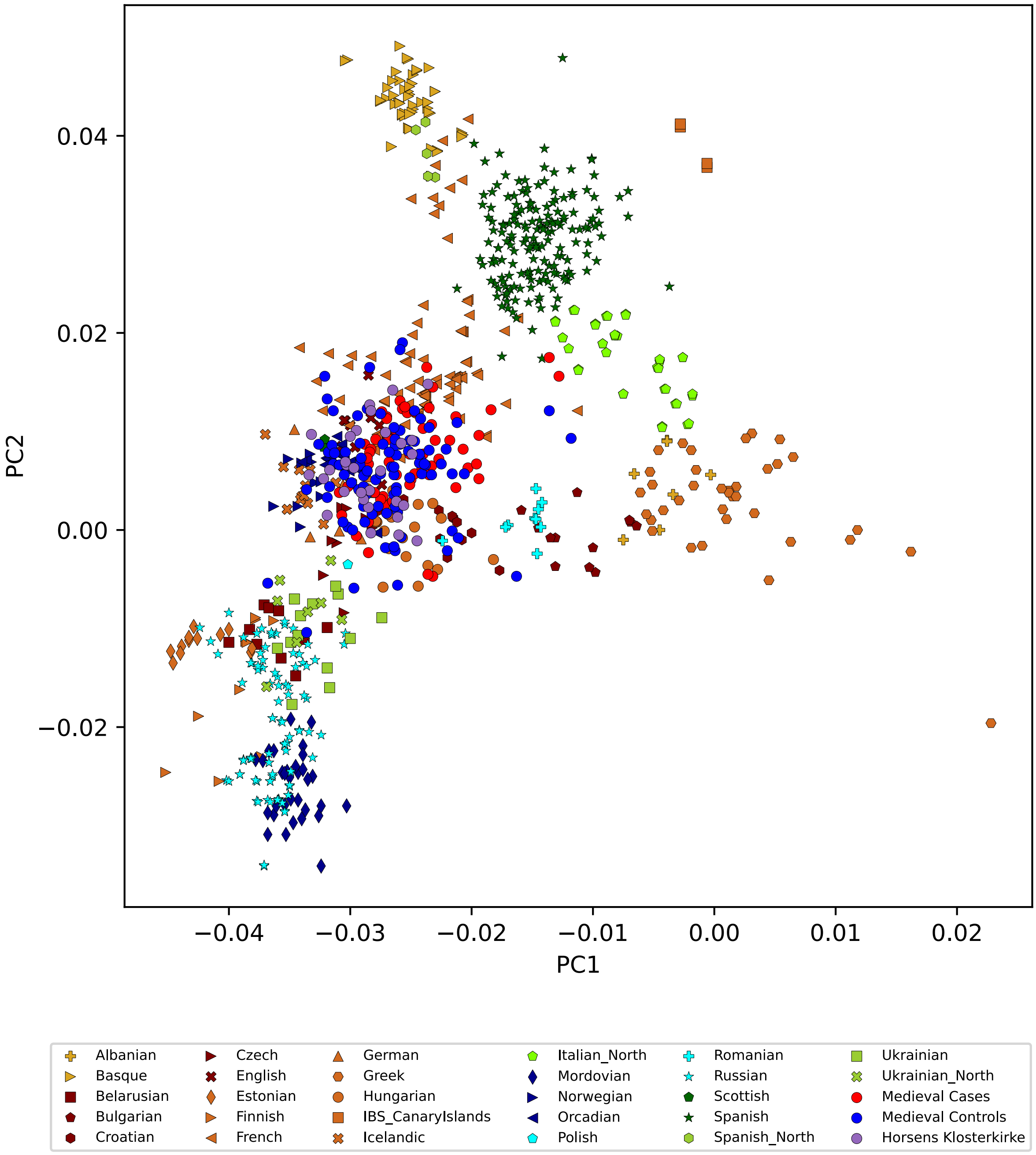

Figure S5. PCA of 58 cases (red circles), 30 individuals from Klosterkirke (purple circles) and 90 remaining controls (blue circles) were projected onto the first two principal components calculated from 66 present-day West-Eurasian populations. For clarity, only the closely-clustering 27 populations were plotted.

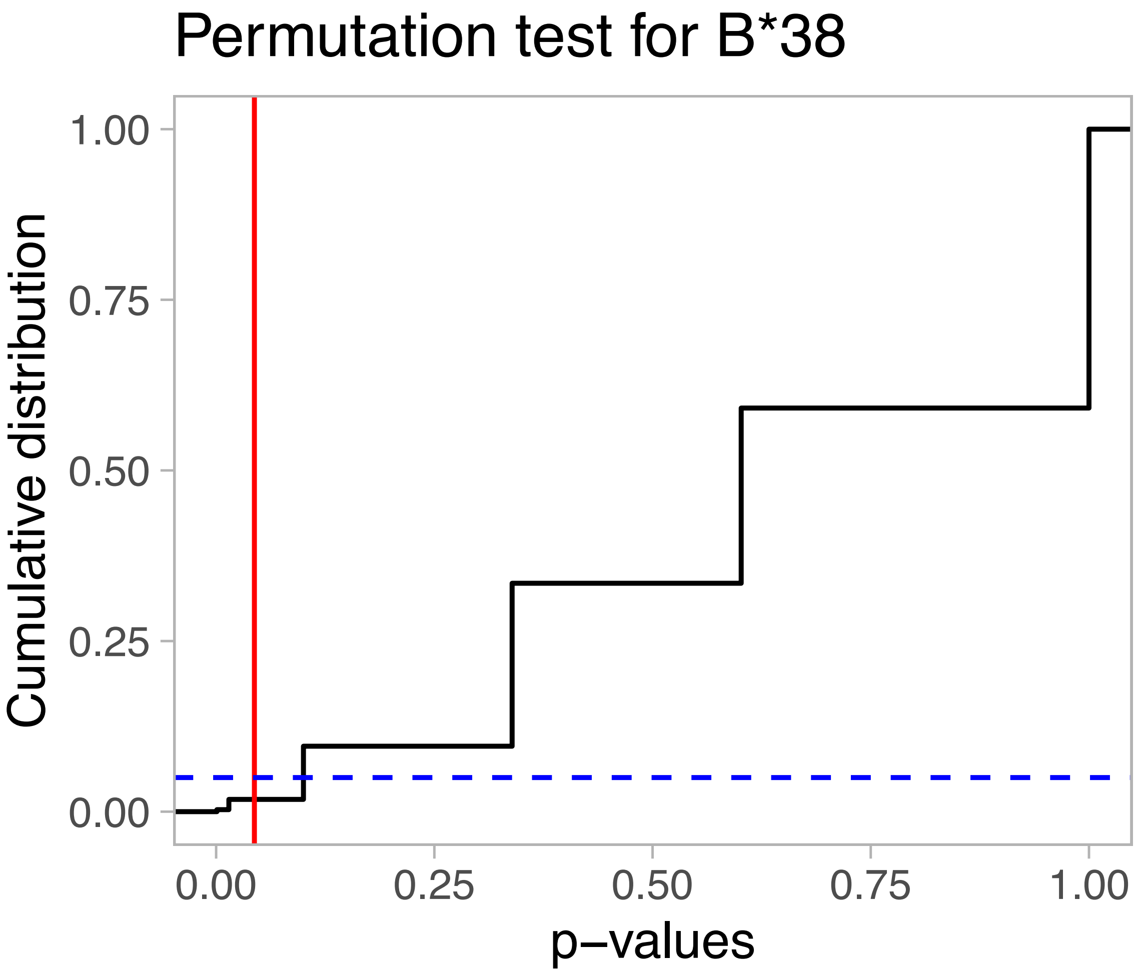

Figure S6. Cumulative distribution of p-values for the B*38 allele obtained by applying Fisher test to 1000 permutations. Red line indicates the observed p-value and the blue line indicates the threshold for 50 / 1000 permutations, corresponding to a significance of 0.05. The observed p-value is below this significance level, supporting that the frequency difference of B*38 between cases and controls is statistically significant.

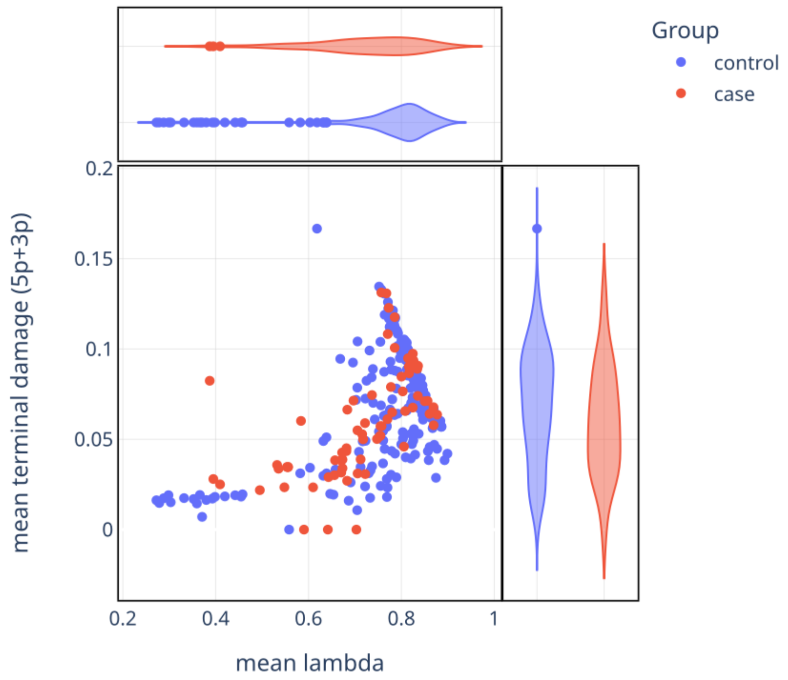

Figure S7. Comparison of DNA damage parameters between case and control groups.

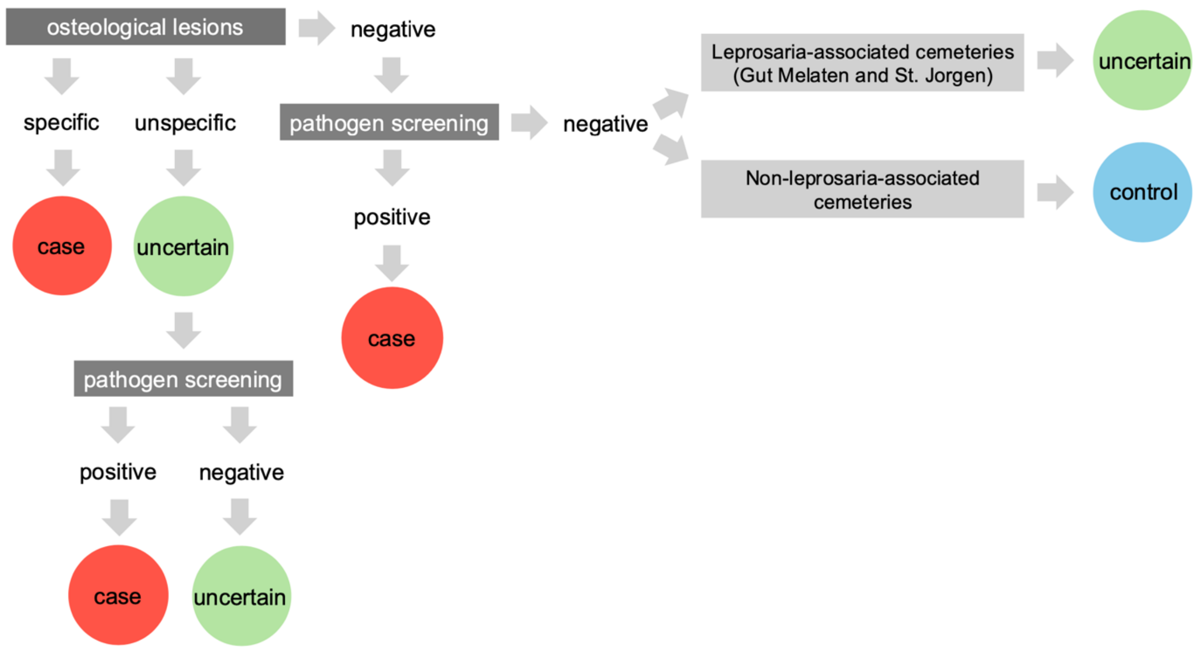

Figure S8. Simplified schematic of the process of disease status determination. Positive results of the paleopathological analysis refer to the presence of lepromatous leprosy (LL) diagnostic lesions as described in the Methods section. Pathogen screening was determined as positive when sequences unique to *M. leprae* were detected in the sample.
