# Supplementary material for: Ancient DNA study provides clues to leprosy susceptibility in medieval Europe
Source: Genome Biol. 2026 Jan 16;27:4. doi: 10.1186/s13059-025-03925-8 (PMC12838506; doi:10.1186/s13059-025-03925-8)
Supplement: Supplementary file 4 — Additional file 4. Review history. [file 13059_2025_3925_MOESM4_ESM.docx]

Review History

**First round of review**

**Reviewer 1**

**Were you able to assess all statistics in the manuscript, including the appropriateness of statistical tests used?**

Yes, and I have assessed the statistics in my report.

**Comments to author:**

Bonczarowska and colleagues examinded in their study entitled "Ancient DNA study provides clues to le 1 prosy susceptibility in medieval Europe" multiple HLA loci in a historical population in the context of leprosy. In addition, they investigated whether any temporal changes in the HLA allele frequencies have occurred in the population since the Middle Ages.
Overall, this is a very interesting and well-made study that deserves in my opinion after some minor modification publication in Genome Biology.
I have the following more specific comments:

Please reorder Figure 1 and Figure 2. Figure 2 should come first and is important to introduce the site and samples and then there should come the current Figure 1 with the first results.
Line 30: Please avoid the expression "for the first time". Especially since some of you already analysed HLA in ancient individuals in the context of leprosy in a previous study (Krause-Kyora 2018).
Line 106+467: Please describe your molecular screening process more in detail. What are the cutoffs for a positive M. leprae cases? You describe 6 M. leprae 6 genomes (> 50% 1X), but 72 samples were used as cases. Are the other 66 cases based on the palaeopathological analysis?
Line 109: Is a genome successfully reconstructed with only 50% covered at 1X? This is a bit optimistic. Please town down.
Line 111: Please include more details to the phylogenetic analysis (type of analysis, sites included, ..).
Line 153: "the samples were"
Line 167: 1240K SNP capture
Line 170: Figure 3B. Does the PcoA plot only show the group averages?
Line 323: The other alleles and protein prediction were not discussed at all. DRB1*13 was associated with protection against leprosy, but were on the lower end of the number of predicted bound peptides. What are your thoughts on that?
Line 353: is it really "interestingly", since HLA is specifically a part of the immune response and alleles should therefore affect susceptibility to diseases etc? In general, you used 8 times the word "interestingly" throughout the manuscript. Please remove all of them. The study is interesting enough!
Line 378: Sample cohort
Line 386: How did the authors make sure that their genotyping wasn't affected by aDNA damage? Only through the TARGT pipeline? Please expand a bit.
Line 391: Sample cohort
Line 441: The DamageProfiler results are not reported anywhere. In general, the authentication of aDNA is not really presented.
Line 444. Insert (Tab S1).
Line 453: underscore between two sentences instead of a space.
Line 597: The data under Accession Number PRJEB66169 is not yet available.
Other comments: Was it at all possible to discern disease severity on affected remains?

**Reviewer 2**

**Were you able to assess all statistics in the manuscript, including the appropriateness of statistical tests used?**

Yes, and I have assessed the statistics in my report.

**Comments to author:**

Review of Bonczarowska et al.

I applaud the authors for venturing into this unexplored and interesting realm of susceptibility and protective alleles driven by infectious disease burden of the past.
While I encourage the publication of this manuscript eventually, there are currently several missing pieces that would make it a much stronger and hence credible paper.
Major Points:
The authors rely entirely on the capture and enrichment of HLA alleles from a set of individuals deemed cases and controls. There are several points that require further clarification on these fronts.
1. What was the overall state of preservation for each sample (case and control). The authors shotgun sequenced each sample and claimed that samples that had ~1% endogenous human DNA were subsequently used for enrichment. But certainly that value ranged dramatically and should be elucidated carefully in a full table.
2. The use of osteological, molecular and archaeological evidence to rule in or out cases vs controls is unclear. Why weren't all samples from the Leprosoria consider positive cases? From those sites how many samples had no osteological, molecular and archaeological evidence to suggest that they weren't cases that were simply too degraded, had died of other co-infecting pathogens (quite a lot circulating at these times). Can the authors redo the calculations in which case all samples at known leprosoria are used as cases not both cases and controls? (I may have mis represented your data here-as I couldn't find the exact number from the tables - if so apologies).
3. Does the preservation of endogenous DNA in the case vs control samples differ at all? Are they statistically different? If they are different, how might this affect the calls being made for both ancestry and HLA diversity.
4. There are NO statistics on coverage depth for any of the SNPs used in the population genetic analyses? The authors claim they covered ~1M SNPs to infer ancestry, yet they provide no coverage of the depth of each SNP.
5. The same critique follows the HLA data. Provide the baits used to target the few HLA alleles the authors discuss? Are they normalized to GC, length etc? Was there a bias in enrichment that suggested some enriched better than others? Did the coverage depth of enrichments align with the depth generated for the SNPs used in population genetic analyses? Much more work on the enrichment and shogun data must be analyzed and provided for a clear cross comparison. A good sample should be good for shotgun and for enrichment? Was this the case? Please provide clear tabulated data. I nice supplementary figure of the coverage depth per sample per allele would be handy to visualize the data.
6. The authors did not perform UDG treatment of samples, and this can lead to serious issues in allele calling. As such the authors claim that by removing a single base at the 5 and 3' ends they are 'removing' damage signal (lines 442-443). But that's not the case. Damage is spread across both termini and depending on how damage each sample is they can affect allele calling dramatically. I emplore the authors to extract mean lambda values from the Bayesian estimation of damage characteristics (using MapDamage 2.0), which describes the per-base probability of terminating an overhang creating a geometric distribution of overhang lengths. You can use the geometric distribution to calculate the overhang length which encompasses 95% of inferred overhangs per value of lambda. Then cleave those away (at various levels, 50%, 90%, 95%) and re-map to see how the damage signal drops and which SNPs remain and aren't likely caused by the damage signal. A retesting of the frequencies and population genetic estimates before and after would be helpful to ensure that frequency data hasn't been overestimated. (Can the authors add the damage profiles for the samples as supplemental information)?
7. The samples have age ranges of 500 years. Have the authors accounted for non-contemporality of the samples? This would drastically affect changes in frequencies over time, if the age of each sample can't be clearly accounted for. If the authors have - I suggest a clear figure here showing the estimated age ranges for the samples and presumed allelic diversity. This will allow for a careful test (statistical) of differences in frequencies per age to see if that is clear.
8. Line 115 - The genomes of the M. leprae are clonal and have little to no diversity. While I have no doubt about their authenticity in this case, the low coverage of several of them leaves doubts about the statement "considerable degree of diversity among them"- suggest alternative language.
9. Table 3 have the authors tested the significance of drawing randomly the same case and control numbers? And importantly in the cases, is the allelic diversity (richness) a factor of a few individuals or random across the entire 83 (especially where there is a difference)?
10. If there are a few individuals who harbour a disproportionate amount of the diversity, I would be weary of damage related artefacts - see point 6. Please check.
11. Given the high differentiation in a few alleles namely B*44 and DRBI*15. It would be helpful to compare the bait set designed for the alleles and how the reads mapped across (again depth and the coefficient of variation) would be helpful here.
12. Same for A*23. They have 7 described alleles in the cases vs 1 in the control. Can the authors detail the information on depth, coverage of each allele, percent covered or each novel allele and the coefficient of variation across each allele?
13. Lines 262- It is very unclear if there is indeed significance in the temporal distribution of the alleles at all, given the large date ranges for each archaeological site. The authors must do an analysis on the overlap of these sites and whether that affects their estimates of change over time. It is extremely difficult to get proper age controls on samples given CI for C14 dating or worse the effects of marine reservoirs in certain locations.
14. It is very unclear what the power of the binding predictions bring to this paper. While I appreciate where the authors are headed, the phrases "binds the lowest number of peptides", or "the average number of peptides leave little in way of discernment as to their effectiveness in functionality. Unless a more rigorous analyses can be performed, suggest removing for clarity of the manuscript.

Minor Points:
Abstract line 31: 18 archaeological sites but n=16.

**Reviewer 3**

**Were you able to assess all statistics in the manuscript, including the appropriateness of statistical tests used?**

Yes, and I have assessed the statistics in my report.

**Comments to author:**

The manuscript by Bonczarowska and colleagues presents a case-control analysis of HLA alleles and leprosy in a population of medieval Europeans. These include 293 individuals from archaeological sites in Germany and Denmark, of which 83 were determined to have leprosy either through osteological analysis or molecular screening of M. leprae DNA. The authors found that HLA B*38 was associated with increased risk of leprosy; this allele has been previously linked to risk of leprosy in modern-day non-European populations. The authors also found novel variants including HLA-A*23 and DPB1*452, which likely increase susceptibility to leprosy, and DRB1*13, which likely confers protection from leprosy in the medieval population. Overall, the study highlights the potential of conducting case-control association analysis in ancient human populations to estimate genetic predisposition to diseases in the past.

The manuscript is well-written, the methodologies and analyses seem technically sound, and the authors do a decent job of mentioning the limitations of the study (e.g. it is underpowered). While it is interesting that case-control studies can be carried out in ancient populations, the impact of the present study seems limited to me.

A few minor comments are given below:

The leprosy cases in this study all pre-date 1600 CE. However, numerous control-group individuals, including all 49 individuals from the site of Horsens Klosterkirke, date from 1600 to 1800 CE. Is it appropriate to include these individuals in the control group, given that they are not contemporaneous to the cases?

Line 51: M. lepromatosis should also be cited as an overall causative agent of leprosy.

Line 453: Remove "_" before the word "subsequently".

Table 4: Please check proper spacing and formatting of table columns.

**Authors Response**

**Point-by-point responses to the reviewers’ comments:**

We sincerely thank the three reviewers for their valuable insights and constructive comments, which have greatly enhanced our manuscript. We have carefully addressed each of their questions and suggestions in the following point-by-point response.

**Reviewer 1 – comments**

1. Please reorder Figure 1 and Figure 2. Figure 2 should come first and is important to introduce the site and samples and then there should come the current Figure 1 with the first results.

We have followed your suggestion to introduce sites and samples on a map and added an additional figure. To streamline the results more efficiently we also divided Table 1 into two: Table 1 and Table 3. Now Figure 1 and Table 1 introduce the reader to the sites and the samples. Table 3 and Figure 3 show the results of case-control status determination.

2. Line 30: Please avoid the expression "for the first time". Especially since some of you already analysed HLA in ancient individuals in the context of leprosy in a previous study (Krause-Kyora 2018).

The phrase “for the first time” was removed.

3. Line 106+467: Please describe your molecular screening process more in detail. What are the cutoffs for a positive M. leprae cases? You describe 6 M. leprae 6 genomes (> 50% 1X), but 72 samples were used as cases. Are the other 66 cases based on the palaeopathological analysis?

Regarding molecular screening, the positive status was assigned when the screening revealed reads aligned to *M. leprae* reference. The aligned sequences were then blasted (megablast) against the core nucleotide database (core_nt) on the NCBI website and if the sequences were unique to *M. leprae* (did not exhibit high similarity to anything else in the database), the leprosy-positive status was then assigned. Subsequently, a targeted capture approach was applied to enrich these libraries for Mycobacterial DNA and the screening was repeated. There was no cut off value. The lowest number of reads unique to *M. leprae* we registered was 4. We expanded the explanation of the molecular screening process in the manuscript.

Lines 503-508: The aligned sequences were then blasted (megablast) against the core nucleotide database (core_nt) on the NCBI website and the leprosy-positive status was assigned when blast revealed sequences unique to *M. leprae*. There was no cut-off value. The lowest number of reads unique to *M. leprae* we observed was 4. Samples positive for the pathogen were then subjected to targeted in-solution capture using myBaits® from Arbor Biosciences [91] to obtain more data for analysis.

The case status was based on both the screening for *M. lep*rae and paleopathological analysis. As for the pathogen screening, in addition to the six cases mentioned above, there were 33 individuals who tested positive for *M. leprae*. In addition, 36 individuals were diagnosed with leprosy based on specific bone lesions. This information can be found in Additional File 1: Tab. S1 and now also in a new Table 2. We have added a paragraph in the results section to present these numbers.

Lines 129-141: Leprosy was diagnosed in the skeletal remains of 75 individuals from the Gut Melaten and Skt. Jørgensgården cemeteries using a combination of genomic and osteological methods (Tab. 2; Additional file 1: Tab. S1). Metagenomic screening for the presence of *M. leprae* reads revealed nine (15.5%) individuals from Gut Melaten and 30 (44.2%) individuals from the Skt. Jørgensgården cemetery to have been infected. *M. leprae* DNA was not detected at any other site. In addition to individuals positive for the pathogen, 36 individuals (19 from Gut Melaten and 17 from Skt. Jørgensgården) were assigned to the case group based on the presence of skeletal lesions indicative of leprosy. The remaining 51 individuals from the leprosaria-associated cemeteries could not be confirmed as leprosy-affected and their case status was considered uncertain. Subsequently, they were removed from further analysis.

4. Line 109: Is a genome successfully reconstructed with only 50% covered at 1X? This is a bit optimistic. Please town down.

We have toned down the sentence.

Lines 146-147: As an aside, we also reconstructed six partial *M. leprae* genomes (51-97%, 1.5-11.7x), five from Gut Melaten and one from Skt. Jørgensgården (Tab. 3).

5. Line 111: Please include more details to the phylogenetic analysis (type of analysis, sites included, ..).

We have deleted the sentence in the results section but have expanded the Methods section.

Lines 514-518: UnifiedGenotyper module of the Genome Analysis Toolkit (GATK) v3.6 [93] was used to generate a VCF file. A set of 3124 phylogenetically informative SNPs [15] was extracted from the VCF (min. coverage 3x, min quality 30, majority call 90%). The phylogeny was reconstructed with the maximum likelihood algorithm using RAxML [94] and a GTR-GAMMA mod. Bootstrapping of 500 replicates was applied.

6. Line 153: "the samples were"

The sentence was corrected.

7. Line 167: 1240K SNP capture

The spelling mistake was corrected.

8. Line 170: Figure 3B. Does the PcoA plot only show the group averages?

The PCoA plot is based on pairwise FST values between populations (please see Table 1 below). Therefore, there is a single value for each pair of groups in the dataset. These values were used as a distance matrix for the PCoA calculations.

**Table 1**. Pairwise FST values between populations.

GBR	FIN	IBS	CEU	TSI	control	case

GBR	0.000	0.011	0.006	0.000	0.010	0.005	0.002

FIN	0.011	0.000	0.018	0.015	0.020	0.017	0.016

IBS	0.006	0.018	0.000	0.011	0.003	0.016	0.017

CEU	0.000	0.015	0.011	0.000	0.016	0.008	0.003

TSI	0.010	0.020	0.003	0.016	0.000	0.018	0.018

control	0.005	0.017	0.016	0.008	0.018	0.000	0.005

case	0.002	0.016	0.017	0.003	0.018	0.005	0.000

We expanded the Methods section to provide more details about the PCoA plot.

Lines 600-608: HLA genotype data of five European populations from the 1000 Genomes Project were incorporated in the analysis of genetic differentiation between medieval cases and controls as well as between the medieval sample and the present-day populations [108]. The modern populations used were Utah residents (CEPH) with Northern and Western European ancestry (CEU), the Iberian population from Spain (IBS), the Toscani from Italy (TSI), populations of Finland (FIN) as well as England and Scotland (GBR). Pairwise Weir-Cockerham F_ST_ estimations based on individuals with complete HLA-A, B, C, DRB1 and DQB1 genotypes at the first-field resolution were obtained with the *adegenet* and *hierfstat* packages in R v. 4.3.1 [109]. Pairwise FST values were used as a distance matrix for the calculations of the principal coordinates analysis (PCoA).

9. Line 323: The other alleles and protein prediction were not discussed at all. DRB1*13 was associated with protection against leprosy, but were on the lower end of the number of predicted bound peptides. What are your thoughts on that?

Following the reviewer’s comment, we focused more on the associated alleles in the Results and Discussion sections.

Results:

Lines 304-311: Among the alleles that were observed at significantly different frequencies in cases and controls for both association analyses, HLA-A*23:01 (Fig. 5A and B), -B*44 alleles (Fig. 5C and D) and -DRB1*15:01 (Fig. 5E and F) are placed on the lower end of the spectrum. While HLA-B*38:01 deviates from this trend, it still binds relatively few peptides, especially when considering strong binders (Fig. 5D). The potentially protective HLA-DRB1*13 allele family is represented by two alleles, -DRB1*13:01 and -DRB1*13:02. Although they differ markedly in terms of the number of weakly bound peptides, both are placed above average when focusing on strongly bound peptides (Fig. 5F).

Discussion:

Lines 370-382: The presentation of specific peptides on the cell surface is thought to influence HLA-mediated resistance or susceptibility to diseases. When the number of *M. leprae*-derived peptides bound by the HLA-A, -B and -DRB1 alleles was analyzed, we noted that the (potential) risk alleles (A*23, B*38, B*44, DRB1*15) tend to bind fewer peptides (Fig. 5). This was particularly apparent for A*23 that is among the HLA-A variants exhibiting the smallest peptide repertoire (Fig. 4A and B). In contrast, the two common alleles (i.e., DRB1*13:01 and DRB1*13:02) for HLA-DRB1*13, which was nominally associated with leprosy protection, bind more than the average number of peptides (Fig. 5E). The number of bound *M. leprae* peptides could potentially affect the efficiency of antigen recognition and contribute to the association the alleles and leprosy susceptibility/protection. However, it should be noted that the number of HLA-presented peptides is not the only determinant influencing the immune response. There are likely other factors involved in the recognition and reaction to *M. leprae* that we could not account for in this study, such as expression levels of antigenic peptides or immunodominance [70].

10. Line 353: is it really "interestingly", since HLA is specifically a part of the immune response and alleles should therefore affect susceptibility to diseases etc? In general, you used 8 times the word "interestingly" throughout the manuscript. Please remove all of them. The study is interesting enough!

Thank you for pointing this out. The manuscript was adjusted according to the reviewer’s comments.

11. Line 378: Sample cohort

The sentence was corrected.

12. Line 386: How did the authors make sure that their genotyping wasn't affected by aDNA damage? Only through the TARGT pipeline? Please expand a bit.

We have applied half-UDG treatment to the libraries which reduces the deamination to the first and last couple of bases. In addition, we used clip and merge to trim the affected bases at the beginning and the end of a sequence. These procedures minimize the aDNA damage.

To reduce the influence of the remaining little aDNA damage on the typing, we employed the pipeline that is, currently, the most suitable for such data (TARGT), and an additional line of evidence for the class I calls was obtained using Optitype. As the allele typing using TARGT is manual, it was possible to visually exclude reads with single substitutions that did not match most of the reads aligned to a particular locus (likely artifacts). An example is shown below:

REFERENCE: AACTCTAGCTAGCTGTTCAGACTGAGAATCATCATCGGAT

Read1: AACTCTAGCTAGCTGTTCAGACTG

Read2: CTAGCTGTTCAGACTGAGAATCAT

Read3: TCGCTGTTCAGACTGAGAATCATCATCGGAT

Read4: GCTGTTCAGACTGAGAATCAG

Read5: AGACTGAGAATCATCATCGGAT

In addition, the approach for the manual calling was strict, i.e., the allele was not called unless there was 100% identity between the reference and the consensus of the aligned sequences. No assumptions were made when HLA typing.

We expanded on this topic in the Discussion section.

Lines 443-451: Second, errors in genotyping could have theoretically influenced the HLA frequencies. The highly degraded nature of aDNA makes the HLA typing challenging. The semi-automated TARGT pipeline is currently the most suitable method for this kind of data [41]. To limit genotyping errors, the approach for the manual calling was strict, i.e., the allele was not called unless there was 100% identity between the reference and the consensus of the aligned sequences. No assumptions were made when HLA typing. Furthermore, Optitype [77] was used to support or reject a call for class I alleles where the manual call was ambiguous. Lastly, this study was mostly explorative. Whilst such analysis can detect potentially novel associations, as we did here, confirmation of the findings in an independent ancient cohort from Europe is necessary.

13. Line 391: Sample cohort

The sentence was corrected.

14. Line 441: The DamageProfiler results are not reported anywhere. In general, the authentication of aDNA is not really presented.

Deamination rates are now reported in Table S1.

15. Line 444. Insert (Tab S1).

We referred to Table S1 as pointed out by the reviewer.

16. Line 453: underscore between two sentences instead of a space.

Corrected.

17. Line 597: The data under Accession Number PRJEB66169 is not yet available.

The data will be available to the public once the manuscript is accepted for publication. However, we can share the data with the reviewer if requested.

18. Other comments: Was it at all possible to discern disease severity on affected remains?

All individuals showing lesions on the skeleton that are characteristic enough for us to make a differential diagnosis of leprosy likely had the severe form of lepromatous leprosy. Individuals only positive for *M. leprae* DNA without any lesions could indicate a milder leprosy form or a severe leprosy form in its early stages. Since leprosy is chronic and may take many years to affect the skeleton, it is not possible to tell whether the disease would eventually manifest as severe on the skeleton if the person lived longer.

**Reviewer 2 – comments**

1. What was the overall state of preservation for each sample (case and control). The authors shotgun sequenced each sample and claimed that samples that had ~1% endogenous human DNA were subsequently used for enrichment. But certainly that value ranged dramatically and should be elucidated carefully in a full table.

Endogenous DNA content from shotgun sequencing was included in Table S1.

2. The use of osteological, molecular and archaeological evidence to rule in or out cases vs controls is unclear. Why weren't all samples from the Leprosoria consider positive cases? From those sites how many samples had no osteological, molecular and archaeological evidence to suggest that they weren't cases that were simply too degraded, had died of other co-infecting pathogens (quite a lot circulating at these times). Can the authors redo the calculations in which case all samples at known leprosoria are used as cases not both cases and controls? (I may have mis represented your data here-as I couldn't find the exact number from the tables - if so apologies).

To achieve the highest statistical power possible in such dataset, the selection criteria for the case group were strict. We could not assume all individuals buried at the leprosaria-associated cemeteries suffered from leprosy because leprosaria did not house exclusively individuals with leprosy. These institutions were known to also provide housing and care for the sick and the poor. Furthermore, as the leprosy prevalence started to drop in the late Middle Ages, leprosaria were usually turned into general infirmaries. It is thus likely that not all individuals buried at these cemeteries suffered from leprosy. Only individuals for which we had molecular evidence of leprosy (*M. leprae* DNA) and/or pathognomonic for leprosy skeletal lesions were considered as cases. Including individuals who may not have had leprosy in the case group could skew the results and were for this reason deliberately removed as part of our selection approach.

The section of the Methods and Results describing the criteria and selection process was clarified and a new table with the exact numbers (Tab. 2) was added to the manuscript. Information about the disease status and the criteria applied are now found in Additional file 1: Table S1.

Results:

Lines 129-141: Leprosy was diagnosed in the skeletal remains of 75 individuals from the Gut Melaten and Skt. Jørgensgården cemeteries using a combination of genomic and osteological methods (Tab. 2; Additional file 1: Tab. S1). Metagenomic screening for the presence of *M. leprae* reads revealed nine (16.1%) individuals from Gut Melaten and 30 (44.2%) individuals from the Skt. Jørgensgården cemetery to have been infected. *M. leprae* DNA was not detected at any other site. In addition to individuals positive for the pathogen, 36 individuals (19 from Gut Melaten and 17 from Skt. Jørgensgården) were assigned to the case group based on the presence of skeletal lesions indicative of leprosy. The remaining 49 individuals from the leprosaria-associated cemeteries could not be confirmed as leprosy-affected and their case status was considered uncertain. Subsequently, they were removed from further analysis. Out of 228 individuals from the remaining cemeteries, 226 were assigned the control status based on the absence of genetic and/or osteological evidence of leprosy (Tab. 1). The disease status of two individuals from Tirup kirke was considered uncertain and these individuals were excluded from the analysis.

Methods:

Lines 534-545: For the leprosaria-associated cemeteries (Skt. Jørgensgården and Gut Melaten), individuals who exhibited skeletal lesions pathognomonic for leprosy and/or molecular evidence of infection were treated as “cases”. As both leprosaria were eventually transformed into either a general infirmary (Gut Melaten) [40,96] and a cemetery for the poor (Skt. Jørgensgården) [97], it is possible that leprosy-unaffected individuals were among the people buried at the associated cemeteries. Therefore, the disease status for the individuals who showed neither molecular nor osteological signs of leprosy was considered uncertain, and they were excluded from further analysis.

Individuals from the remaining cemeteries were assigned the “control” status based on the absence of molecular and/or osteological evidence of leprosy. Two individuals from Tirup Kirke, who showed unspecific skeletal lesions suggestive of a systemic infectious disease, were excluded from the analysis due to the uncertain disease status.

3. Does the preservation of endogenous DNA in the case vs control samples differ at all? Are they statistically different? If they are different, how might this affect the calls being made for both ancestry and HLA diversity.

The preservation of aDNA naturally varies among samples and sites. In our data we can observe variation in the preservation depending on the material from which aDNA was extracted (Fig. 1 below). However, overall, we did not observe significant differences in endogenous aDNA between cases and controls (Fig. 2 below).

**Figure 1.** Endogenous DNA content varies among different sample types. Petrous bones (PB) are usually better preserved than teeth (T).

**Figure 2.** Endogenous DNA between cases and controls. The p-value displayed was obtained from a two-sided Mann-Whitney U test.

To reduce the influence of the aDNA damage on the HLA typing, please see our comment above (Reviewer 1, comment 12).

For population genetics analysis, all individuals included in the analysis met a threshold of 20,000 SNPs which is commonly used in ancient population genetic studies (e.g. Sirak et al., 2021 *https://doi.org/10.1038/s41467-021-27356-8*). Sequence data preprocessing and subsequent analysis were done according to current methodological standards developed for ancient metagenomic data. We can appreciate the limitations inherent to ancient DNA and openly presented them in the Discussion section (Lines 437-451).

4. There are NO statistics on coverage depth for any of the SNPs used in the population genetic analyses? The authors claim they covered ~1M SNPs to infer ancestry, yet they provide no coverage of the depth of each SNP.

We have expanded Table S1 with additional information from the population genetic analyses, including detailed statistics on mapping quality relative to the human reference genome (hg19) and the number of genotyped SNPs per sample.

5. The same critique follows the HLA data. Provide the baits used to target the few HLA alleles the authors discuss? Are they normalized to GC, length etc? Was there a bias in enrichment that suggested some enriched better than others? Did the coverage depth of enrichments align with the depth generated for the SNPs used in population genetic analyses? Much more work on the enrichment and shogun data must be analyzed and provided for a clear cross comparison. A good sample should be good for shotgun and for enrichment? Was this the case? Please provide clear tabulated data. I nice supplementary figure of the coverage depth per sample per allele would be handy to visualize the data.

The HLA capture probes were designed as described by Wittig et al., 2015; *https://pubmed.ncbi.nlm.nih.gov/25753671/*. Information about baits, design and performance with modern DNA is provided in the Wittig et al. publication. A benchmarking test for the HLA capture and the calling pipeline for ancient DNA is published in Pierini et al. 2020 [41].

This information was added in the Methods section.

Lines 564-565: One sample per individual was subjected to targeted enrichment of the HLA exonic regions using a bait panel designed previously [104].

The panel has been tested and successfully applied to ancient data (e.g., ref. 17, 46, 41, 59, 78,79). HLA typing and genotyping was done in a strict manner using the most appropriate tools as of now (please see the response to comment 3). In the authors' opinion, showcasing coverage depth for each allele for all the samples does not explain the results presented in the study. However, additional information about endogenous DNA content, deamination, and mapping statistics were added in the supplementary Table S1.

As for the requested cross comparison between HLA enrichment and shogun data (for population genetics), we show in Fig. 3 (below), a correlation between the coverage generated with HLA enrichment and the SNP coverage prior to enrichment.

**Figure 3.** Correlation between the coverage of SNPs from the 1240K panel (used for population genetic analyses and obtained via shotgun sequencing) and the coverage on HLA loci (obtained through enrichment/capture). The X-axis shows the number of SNPs genotyped in the 1240K panel (pseudo-haploid genotypes). The Y-axis represents the mean depth of the enriched region for each locus: exons 2 and 3 for the HLA class I loci (HLA-A, HLA-B, and HLA-C), and exon 2 for the HLA class II loci (HLA-DPB1, HLA-DQB1, and HLA-DRB1). Pearson’s correlation coefficient (r) and p-value are displayed for each locus.

6. The authors did not perform UDG treatment of samples, and this can lead to serious issues in allele calling. As such the authors claim that by removing a single base at the 5 and 3' ends they are 'removing' damage signal (lines 442-443). But that's not the case. Damage is spread across both termini and depending on how damage each sample is they can affect allele calling dramatically. I emplore the authors to extract mean lambda values from the Bayesian estimation of damage characteristics (using MapDamage 2.0), which describes the per-base probability of terminating an overhang creating a geometric distribution of overhang lengths. You can use the geometric distribution to calculate the overhang length which encompasses 95% of inferred overhangs per value of lambda. Then cleave those away (at various levels, 50%, 90%, 95%) and re-map to see how the damage signal drops and which SNPs remain and aren't likely caused by the damage signal. A retesting of the frequencies and population genetic estimates before and after would be helpful to ensure that frequency data hasn't been overestimated. (Can the authors add the damage profiles for the samples as supplemental information)?

The authors want to apologize for the confusion. Indeed, in the first draft of the manuscript, we erroneously stated that we used non-UDG protocols. However, we applied **half-UDG** treatment to all samples. This mistake was corrected in the Methods section of the manuscript.

Lines 481-483: All DNA samples were extracted and processed in a dedicated aDNA facility at the University of Kiel following the guidelines on contamination control in ancient DNA [81-83], using a previously published protocol for the **half-UGD** library preparation [17].

Furthermore, the authors stated in the Method section of the manuscript that two positions from each end were trimmed, not one.

Lines 494-495: After the validation, the first two positions from the 5’ and 3’-ends of the reads were trimmed.

As for the allele calling, the authors addressed the reliability and accuracy of both HLA allele typing and genotyping in comments above. Please see responses to comments 3 and 6. Any limitations are discussed in the Discussion section. As damage was treated during both the wet lab and computational processing of the reads, the reviewer’s suggestion of additional analyses are not necessary in the authors’ opinion. Deamination rates are now presented in Table S1.

7. The samples have age ranges of 500 years. Have the authors accounted for non-contemporality of the samples? This would drastically affect changes in frequencies over time, if the age of each sample can't be clearly accounted for. If the authors have - I suggest a clear figure here showing the estimated age ranges for the samples and presumed allelic diversity. This will allow for a careful test (statistical) of differences in frequencies per age to see if that is clear.

Unfortunately, precise dating for each individual is not available and time frames assigned to each location were based on archaeological data. Furthermore, dating ranges overlap between various archaeological sites and thus, it is not possible to accurately divide the sample into temporal subgroups. This issue is now brought up in the Discussion section.

Lines 418-421: It is, however, imperative to mention that the medieval control group includes individuals who lived and died in a period spanning several centuries. It is thus possible that selection varied at different time points [72], as epidemics of various infectious diseases swept across the continent.

8. Line 115 - The genomes of the M. leprae are clonal and have little to no diversity. While I have no doubt about their authenticity in this case, the low coverage of several of them leaves doubts about the statement "considerable degree of diversity among them"- suggest alternative language.

Alternative language was used to describe the phylogenetic placement of Melaten strains.

Lines 147-149: In a phylogenetic tree, all new genomes clustered within the diversity of branch 3 (Fig. 2). The strains from Melaten fell on separate subbranches.

9. Table 3 have the authors tested the significance of drawing randomly the same case and control numbers? And importantly in the cases, is the allelic diversity (richness) a factor of a few individuals or random across the entire 83 (especially where there is a difference)?

In Table 3 (now Table 5), the only significant result is B*38. Following the reviewer’s question, one of the authors performed a permutation test for this allele (Additional file 2: Fig. S5). Briefly, this is done by shuffling the case-control labels. This, presumably, breaks the association as cases become independent of the alleles of an individual. After the shuffling, the Fisher test p-value is calculated for the B*38 allele between cases and controls. This is repeated 1000 times to obtain a distribution of p-values and finally, we check where our original p-value lies within this distribution. The observed p-value was located at the bottom 5%. Therefore, we can assume that the original significant p-value is not a result of sampling bias (i.e. just by chance, all B*38s ended up in “case” group).

We added this result and the related methods explanation into the manuscript.

Lines 242-246: This association was further confirmed using simulations where case-control labels were randomly permuted within the dataset (1000 permutations). Fewer than 5% of permuted p-values matched or exceeded the observed p-value, indicating that the observed difference in B*38 allele frequency is unlikely to have occurred by chance.

Lines 592-597: A permutation test was performed for alleles that showed a significant difference between cases and controls by randomly shuffling case-control labels 1,000 times. For each permutation, the p-value of Fisher's exact test was calculated to generate a null distribution of p-values. The frequency difference between cases and controls was considered significant if the observed p-value fell within the lowest 5% of the null distribution.

Allelic richness is calculated using the allele frequency data for the entire population. This can be affected by a few individuals only if all the rare alleles are observed in those few. This is not the case for our dataset. As expected, rare alleles are found in a heterozygous state, together with a common allele, both for cases and controls. Therefore, it is unlikely that our observations would be biased by a few individuals.

10. If there are a few individuals who harbour a disproportionate amount of the diversity, I would be weary of damage related artefacts - see point 6. Please check. Given the high differentiation in a few alleles namely B*44 and DRBI*15. It would be helpful to compare the bait set designed for the alleles and how the reads mapped across (again depth and the coefficient of variation) would be helpful here.

No, this is not the case. Please see for more details our response to comments 3 (regarding preservation and calling parameter), 5 (regarding bait design and efficiency) and 6 (regarding damage related artefacts). The efficiency of the HLA baits and the evenness of the enrichment was taken into consideration by Wittig et al. when designing the capture.

11. Same for A*23. They have 7 described alleles in the cases vs 1 in the control. Can the authors detail the information on depth, coverage of each allele, percent covered or each novel allele and the coefficient of variation across each allele?

Generally, the genotype rate (n of alleles called/number of samples captured $\times$2) was similar for all loci between cases and controls (Tab. 2 and 3 below) with ~80% for HLA A, B, C, DRB1 and DQB1 and ~40% for DPB1.

**Table 2.** Genotype rate per HLA locus in case and control groups.

| **Group** | **Locus** | **Genotype rate** | **N of alleles called** |
| --- | --- | --- | --- |
| **cases** | HLA-A | 0.853333 | 128 |
|  | HLA-B | 0.746667 | 112 |
|  | HLA-C | 0.813333 | 122 |
|  | HLA-DRB1 | 0.82 | 123 |
|  | HLA-DQB1 | 0.84 | 126 |
|  | HLA-DPB1 | 0.34 | 51 |
| **controls** | HLA-A | 0.833333 | 370 |
|  | HLA-B | 0.813063 | 361 |
|  | HLA-C | 0.792793 | 352 |
|  | HLA-DRB1 | 0.777027 | 345 |
|  | HLA-DQB1 | 0.783784 | 348 |
|  | HLA-DPB1 | 0.459459 | 204 |

**Table 3.** Comparison of genotype rates per locus between cases and controls.

| **Locus** | **Fisher p-value** | **corrected p-value (BH-FDR)** |
| --- | --- | --- |
| **HLA-A** | 0.6098 | 0.6391 |
| **HLA-B** | 0.1002 | 0.3005 |
| **HLA-C** | 0.6391 | 0.6391 |
| **HLA-DRB1** | 0.2993 | 0.4489 |
| **HLA-DQB1** | 0.1584 | 0.3168 |
| **HLA-DPB1** | 0.0130 | 0.0778 |

Following authors’ responses to HLA typing-related comments 3, 5 and 6, the authors would like to add that the proof-of-concept TARGT article [41] showed that while higher coverage increases the rate of genotyping success, even a few reads are sufficient for accurate typing of HLA alleles. That is thanks to the manual part of the pipeline, in which reads are manually curated. Furthermore, Optitype that was used for cross validation of HLA class I allele calls was shown to perform better on low-coverage data relative to other automated pipelines (Thuesen et al., 2022, [*https://doi.org/10.3389/fimmu.2022.987655*](https://doi.org/10.3389/fimmu.2022.987655)). As previously mentioned, this HLA genotyping approach was previously applied in multiple aDNA studies [e.g., ref. 17, 46, 41, 59, 78,79].

12. Lines 262- It is very unclear if there is indeed significance in the temporal distribution of the alleles at all, given the large date ranges for each archaeological site. The authors must do an analysis on the overlap of these sites and whether that affects their estimates of change over time. It is extremely difficult to get proper age controls on samples given CI for C14 dating or worse the effects of marine reservoirs in certain locations.

Please see our response to comment 7.

13. It is very unclear what the power of the binding predictions bring to this paper. While I appreciate where the authors are headed, the phrases "binds the lowest number of peptides", or "the average number of peptides leave little in way of discernment as to their effectiveness in functionality. Unless a more rigorous analyses can be performed, suggest removing for clarity of the manuscript.

Following comments from a different reviewer, the Results and Discussion sections on the binding predictions were modified to focus on the alleles associated in this study. Although our analysis cannot account for factors like the level of antigenic peptide expression and immunodominance, we noted a trend in binding predictions. We decided to report these results as it is likely a question that will pop up in the readers’ minds just like it did for us.

We modified the manuscript see for details Reviewer 1 comment 9

14. Abstract line 31: 18 archaeological sites but n=16.

While the total number of sites is 18, 16 of those are located in Denmark and 2 in Germany.

Lines 32-33: The sample comprised 293 medieval individuals from 18 archaeological sites in Denmark (N=16) and Germany (N=2).

**Reviewer 3 – comments**

1. While it is interesting that case-control studies can be carried out in ancient populations, the impact of the present study seems limited to me.

Nowadays, Europe is free of many infectious diseases which used to be common in the Middle Ages. Therefore, the genetic predisposition to these diseases in present-day Europeans cannot be investigated via case-control association studies, due to the virtual lack of cases. Development of the aDNA technology, however, allows us to perform such analyses in past populations. This is the only approach to obtain genetic information about predisposition to leprosy in Europeans, therefore we deem this study meaningful, even if the association findings may seem limited.

2. The leprosy cases in this study all pre-date 1600 CE. However, numerous control-group individuals, including all 49 individuals from the site of Horsens Klosterkirke, date from 1600 to 1800 CE. Is it appropriate to include these individuals in the control group, given that they are not contemporaneous to the cases?

To address this concern, we performed additional statistical analysis comparing the frequencies of all observed HLA alleles between individuals from Klosterkirke and other controls. We observed no significant differences between these two groups. These results are now included in the Supplement (Additional file 1: Tab. S5). Furthermore, in the PCA based on genomic data, individuals from Klosterkirke overlap with other controls and cases, which is now presented in the Supplement as well (Additional file 2, Figure S4). Based on these results, the authors consider including these individuals in the control group as justified.

Lines 102-104: As the Klosterkirke burials postdate both leprosaria-associated cemeteries (Tab. 1), further analysis was performed to ensure that the individuals from this site could be included in the association study (see below).

Lines 212-218: When cases and controls were considered separately, the groups overlapped on the PCA plot along both axes, including the individuals from the later dating Klosterkirke burials (Additional file 2: Fig. S3 and S4). Furthermore, there were no significant differences in HLA allele frequencies between the individuals from Klosterkirke and the remaining controls (Additional file 1: Tab. S5). These results indicate that there is no substantial genome-wide diversity between any of the groups and a comparison of allelic frequencies in a case-control association study is warranted.

3. Line 51: M. lepromatosis should also be cited as an overall causative agent of leprosy.

While the authors appreciate that leprosy can be caused by *both M. leprae* and *M. lepromatosis*, the focus of this study is Europe. In that sentence we were referring to the medieval leprosy epidemic in Europe that, as far as we know, was caused by *M. leprae*. To not confuse the reader, we decided not to mention *M. lepromatosis* in this context. However, we mentioned *M. lepromatosis*, which we used as an outgroup in the phylogenetic tree (Fig. 2), as in infectious agent in America, in the legend of Fig. 2.

4. Line 453: Remove "_" before the word "subsequently".

The space was removed.

5. Table 4: Please check proper spacing and formatting of table columns.

Spacing and formatting of table columns was checked and corrected.

**Second round of review**

**Reviewer 1**

Thank you for addressing all my comments raised during the first revision.

**Reviewer 3**

In this revision, Bonczarowska et al. have done a good job of addressing my comments on the previous version of the article. However, a second reading of the manuscript and clarification on some of the methods from the authors in response to other reviewers' comments have led me to have the following questions that still need to be addressed before I can recommend the article for publication.

Major issues:

If I now understand the Methods correctly, for assigning leprosy case status using the genetic analysis, a sample was considered as a leprosy case if there were ANY reads in the shotgun sequencing that were assigned to M. leprae using a BLAST search. There does not seem to be any e-value cut-off or minimum number of reads threshold used. Can the authors specify how many top hits were considered in the BLAST search?

The authors state that one of the samples had as low as 4 reads assigned to M. leprae; in my opinion, this is not sufficient to consider a sample positive for a pathogen. These reads could be due to mis-mapping, especially hits in conserved regions of the genome, from other mycobacteria (commonly found in the soil and co-present in skeletal remains). Typically, one would expect a threshold of at least 500-1,000 reads being used, these reads should be evenly spaced across the M. leprae genome (no read stacking), and damage patterns should be checked to make sure they look ancient. As far as I can tell from the section "Pathogen screening and phylogenetic analysis of M. leprae", the authors do not mention that they checked damage patterns of M. leprae reads from the shotgun data.

To this end, I am a bit concerned about how the authors are assigning case status based on the genetic analysis. For those samples which also had osteological data supporting the leprosy diagnosis, it should be fine, but there seem to be at least 16 samples which were assigned as cases solely based on the metagenomic screening. I would like to see a Supplementary Table showing the results of the metagenomic screening of the shotgun data for all the samples so that we can see how many M. leprae reads were assigned to each sample. Furthermore, it would be good to see the results of the capture also added to this table (currently, the authors only show the results of the post-capture mapping for the 6 samples which yielded partial genomes).

Line 490: Was seed disabled for BWA mapping as is typically done in aDNA studies?

Lines 507-508: More details are necessary about the M. leprae genome capture, even if that is not the focus of this study. What capture kit was used? Was it a synthetic bait kit or an RNA baits kit; if the latter, what strains were used to create the baits? What were the hybridization temperature and time conditions? How were captured libraries amplified? What platform was used for sequencing?

Line 509: For the M. leprae genome mapping, what parameters were used? Was seed disabled? Was -n still kept at 0.01 (as in case of the human DNA mapping), or was it increased to 0.1 (which is typically done in ancient microbial studies)?

Lines 512-517: Based on the variant calling parameters used and the statistics given in Table 3, strains G91 and G35 really do not have sufficient 3-fold genome coverage to be included in a tree. I think it is fine to say you recovered partial genomes, but given that only 7% and 20% of the genome, respectively, is callable for those two strains, you are incorporating a lot of missing data. At the very least, the authors should exclude those two strains from the tree and see if they get the same topology.

Minor edits:

Line 58: Just a suggestion - change "characteristic" to "diagnostic".

Line 88: Change to "samples".

Line 146: The words "As an aside" seem weird/incorrect to use in this type of writing. Maybe use "Furthermore" instead?

Line 161: Typo - The word "three" is used instead of "tree".

Line 330: Change "the European Middle Ages" to "medieval Europe" or "the Middle Ages in Europe".

Line 357: Spell out MS as multiple sclerosis since it is the first time it is used in the manuscript.

Line 483" Typo - It should be "UDG", not "UGD".

Lines 480-485: No paper is cited to show which DNA extraction protocol was used. If this is the same as Krause-Kyora et al. 2018, this paper should be cited. Secondly, as far as I can tell, there was no half-UDG treatment done in Krause-Kyora et al. 2018 - it seems to be the libraries in that study were either full-UDG treated or non-UDG treated. Please check the correct citation for your half-UDG treatment protocol.

Lines 503-508: Please change the sentence to remove the word "blasted". BLAST should be capitalized and not used as a verb. The sentence should read "The aligned sequences were then used as queries in a MegaBLAST search against the core nucleotide database (core_nt) on the NCBI website and the leprosy-positive status was assigned when the BLAST analysis revealed sequences unique to M. leprae."

Is the study design appropriate to answer the research question (including the use of appropriate controls), and are the conclusions supported by the evidence presented?
No. Further review of the metagenomic screening for cases is necessary to make sure they should be considered as cases. If the authors sufficiently address my concerns regarding this (see comments above), then I have no further issues.

Are the methods sufficiently described to allow the study to be repeated?
No. The capture methodology needs to be described in more details. Some minor details regarding the mapping should also be clarified (see above comments).

Is the use of statistics and treatment of uncertainties appropriate?
Yes.

Is the presentation of the work clear?
Yes.

Are the images in this manuscript (including electrophoretic gels and blots) free from apparent manipulation?
Yes.

**Authors Response**

**Point-by-point responses to the reviewers’ comments:**

We thank the reviewers for feedback, which has substantially improved the quality of our manuscript. Below, we provide detailed responses to each comment. All new changes in the manuscript text are highlighted in orange.

Additionally, we would like to inform you that two new co-authors have been added to the manuscript (D. Myburgh and K. Fuchs), who contributed to the additional *M. leprae* and osteological analyses as well as participated in the review and revision process. Furthermore, due to an increased contribution and responsibility for the study of Prof. Krause-Kyora, he was placed as the senior author. All co-authors agreed to these changes.

In the process of addressing the comments from Reviewer #3, we incorporated more samples in a reproducible and systematic screening for *Mycobacterium leprae* DNA. Inclusion of additional teeth samples resulted in more individuals testing as positive for *M. leprae.* The authors included these individuals in the association analysis, which resulted in a slight change in the results, i.e., HLA-B*38 did not remain significant after Bonferroni correction; however, it was confirmed as associated with leprosy status with the Firth’s logistic regression. In addition, HLA-DRB1*04 appeared as nominally significant. The updated results were discussed in the Discussion section.

**Reviewer #2 comment**

The Board member requests that you implement the following into a further revised version of the manuscript to fully address Reviewer #2's concern related to removing damage signal: integrate signals of damage (such as lambda, suggested by Reviewer #2, or even other damage/fragmentation measurements) into a multivariate or mixed effects model that then also includes the HLA results. This should be done to ensure that sample age, i.e. which is closely mirrored with DNA damage and degradation, are not driving these differences between cases and controls. Please ensure that preservation bias and age across cases and controls isn't driving these signatures.

To ensure that DNA damage in half-UDG HLA-captured libraries did not confound our HLA analysis, we first compared two indicators of DNA preservation between cases and controls: the λ parameter from damage modeling and the mean terminal deamination rate

(average of 5′ and 3′ misincorporation rates) (Figure 1). While Mann–Whitney U tests indicated significant differences between groups (p = 8.04e-05 for A; p = 0.016 for terminal damage), the distributions of both parameters showed substantial overlap.

Figure 1. Comparison of DNA damage parameters between case and control groups.

To further assess whether DNA preservation could bias our results, we incorporated preservation metrics directly into a multivariate model that includes HLA results. We confirmed that after accounting for differences in damage, HLA alleles remain associated with the case-control status.

Lines 282-297 (**results**): In order to assess the potential confounding effect of DNA damage on HLA associations, two indicators of DNA preservation were compared between cases and controls: the A parameter from damage modeling and the mean terminal deamination rate (average of 5′ and 3′ misincorporation rates) (Additional file 2: Fig. S7). While Mann–Whitney U tests indicated significant differences between groups (p = 8.04e-05 for A; p = 0.016 for terminal damage), the distributions of both parameters showed substantial overlap and no clear evidence of systematic skew or group-specific damage patterns. To control for the effect of damage-related confounding, a multivariate model was employed. Case/control status was defined as a binary outcome, with predictor variables including four HLA alleles of interest (HLA-B*38, HLA-A*23, DRB1*04, and HLA-DRB1*13), along with three measures of DNA preservation: the A parameter,

mean terminal deamination rate and the endogenous DNA content. The HLA alleles were selected based on prior analyses. In line with the previous observations, HLA-B*38, HLA-A*23 and DRB1*04 remained associated with the case status, while HLA-DRB1*13 was protective after accounting for DNA damage (Additional file 1: Tab. S10). The odds ratios derived from this model were consistent with those obtained in the unadjusted association analyses, thereby confirming that observed HLA effects were not driven by DNA damage.

Lines 660-670 (**methods**): To assess the potential confounding effects of DNA damage on HLA association results, we applied a multivariate model incorporating three metrics of DNA preservation: endogenous DNA content, the λ parameter from damage modeling, and the mean terminal deamination rate (calculated as the average of 5′ and 3′ misincorporation rates). The damage parameters (λ and deamination rates) were estimated using mapDamage 2.0 [116], based on half-UDG treated, HLA-captured libraries that were used for HLA genotyping. To evaluate whether these preservation metrics influenced HLA associations, we performed Firth’s logistic regression using the R package *logistf* [117]. The binary outcome variable was disease status (case/control), and predictor variables included four HLA alleles (HLA-B*38, HLA-A*23, HLA-DRB1*13 and DRB1*04), along with the three preservation covariates. Three separate models were carried out, each including one of the three HLA alleles in order to assess each allele’s effect independently.

**Reviewer #3: comments**

If I now understand the Methods correctly, for assigning leprosy case status using the genetic analysis, a sample was considered as a leprosy case if there were ANY reads in the shotgun sequencing that were assigned to M. leprae using a BLAST search. There does not seem to be any e-value cut-off or minimum number of reads threshold used. Can the authors specify how many top hits were considered in the BLAST search?

To illustrate the process of the assignment of the case and control status, an additional figure was added in the supplement (Additional file 2: Fig. S8).

With regards to the metagenomic screening, following Reviewer’s questions, the authors applied a more reproducible and systematic approach for detection of *M. leprae* DNA. To assign an individual to the “case” group, at least five reads had to align to *M. leprae* in a competitive mapping of the metagenomic data against a set of Mycobacterial reference genomes (Additional File 1: Tab. S14). These reads were visually inspected in IGV for excessive SNPs and stacking and further confirmed with megablast (E-value < 1e-5). The approach is now explained in the Methods section and the results from MALT, alignment, competitive mapping, and blast can be now found in Additional file 1: Tab. S1.

Lines 542-548 (**methods**): Competitive mapping was also carried out against 13 *Mycobacterium* references and two non-*Mycobacterium* outgroup species (Additional File 1: Tab. S14) to identify and remove non-specific reads, ensuring that only sequences specific to *M. leprae* were retained for analysis. BWA v0.7.15 [91] was used to perform the alignment with parameters typical for aDNA (-n 0.01 and l -300). Additionally, a mapping quality filter of 30 and an identity filter of 99% were applied. Duplicate reads and sequences shorter than 50bp were removed.

Reads that aligned specifically to the *M. leprae* reference after competitive mapping were manually inspected in Integrative Genomics Viewer (IGV) v2.17.1 [97]. Alignments were checked for even read distribution, absence of stacking, and lack of excessive SNPs or insertions. The identity of these reads was further confirmed with BLAST v2.15.0 [98], using a significance threshold of E-value lower than 1e-5. Only those samples that contained at least 5 *M. leprae* reads in competitive mapping were considered as positive for the pathogen’s DNA.

The authors state that one of the samples had as low as 4 reads assigned to M. leprae; in my opinion, this is not sufficient to consider a sample positive for a pathogen. These reads could be due to mis-mapping, especially hits in conserved regions of the genome, from other mycobacteria (commonly found in the soil and co-present in skeletal remains). Typically, one would expect a threshold of at least 500-1,000 reads being used, these reads should be evenly spaced across the M. leprae genome (no read stacking), and damage patterns should be checked to make sure they look ancient. As far as I can tell from the section "Pathogen screening and phylogenetic analysis of M. leprae", the authors do not mention that they checked damage patterns of M. leprae reads from the shotgun data. To this end, I am a bit concerned about how the authors are assigning case status based on the genetic analysis. For those samples which also had osteological data supporting the leprosy diagnosis, it should be fine, but there seem to be at least 16 samples which were assigned as cases solely based on the metagenomic screening.

Following up on the response above, the positive status was only assigned when there were at least five reads aligning to *M. leprae* in a competitive mapping. Furthermore, the reads were analyzed using megablast and all samples considered as leprosy-positive contained at least one read aligning exclusively to *M. leprae* (These results are shown in Additional file 1: Tab. S1.). The genome of *M. leprae* is highly conserved. Only 3124 informative SNPs are necessary for phylogenetic analysis of *M. leprae*. In the authors’ experience, just a few unique reads (that do not exhibit an excessive number of SNPs and stacking in the alignment) are sufficient for detection of this pathogen.

Damage pattern was assessed for six partially reconstructed *M. leprae* genomes shown in Table 3. The results show a typical for aDNA damage pattern, which can be seen in the Additional file 2: Fig. S1.

Lines 147-149 (**results**): The alignment of sequence reads to M. leprae genome demonstrated deamination signatures at terminal positions, supporting the authenticity of their ancient origin (Additional File 2: Fig. S1).

Lines 570-571 (**methods**): The ancient origin of these strains was confirmed by evaluation of terminal read damage with DamageProfiler [92].

I would like to see a Supplementary Table showing the results of the metagenomic screening of the shotgun data for all the samples so that we can see how many M. leprae reads were assigned to each sample.

The results of the MALT screening, alignment against *M. leprae* reference genome, competitive mapping, and megablast can be now found in Additional file 1: Tab. S1.

Furthermore, it would be good to see the results of the capture also added to this table (currently, the authors only show the results of the post-capture mapping for the 6 samples which yielded partial genomes).

The number of *M. leprae* reads generated using targeted capture was added for each captured sample in the supplementary Table S1.

Note: As more samples from Skt. Jørgensgården were added to the study since the submission for the purpose of pathogen screening, not all samples positive for *M. leprae* DNA were captured. The capture efforts were focused on reconstructing bacterial genomes mainly from the Gut Melaten leprosarium. Gut Melaten was previously unexplored in the context of genetic diversity of the leprosy pathogen, in contrast to Skt. Jørgensgården, which was already shown to harbour high diversity of *M. leprae* [(10.1371/journal.ppat.1006997)](https://doi.org/10.1371/journal.ppat.1006997).

Line 490: Was seed disabled for BWA mapping as is typically done in aDNA studies?

Yes, seeding was disabled by setting the seed length parameter to 300 (-l 300). This information was added to the Methods (lines: 527-528).

Lines 507-508: More details are necessary about the M. leprae genome capture, even if that is not the focus of this study. What capture kit was used? Was it a synthetic bait kit or an RNA baits kit; if the latter, what strains were used to create the baits? What were the hybridization temperature and time conditions? How were captured libraries amplified? What platform was used for sequencing?

Additional information about the capture design was added to the Methods section.

Lines 557-563: (**methods**): Samples positive for the pathogen were then subjected to targeted in-solution capture to obtain more data or analysis [99]. Biotinylated 70bp RNA baits (myBaits® from Arbor Biosciences) were designed for *M. leprae* TN strain (NC_002677.1) using 3x tiling. The baits were filtered for low sequence complexity and duplicates, resulting in a set of 135,953 baits. Two rounds of hybridization capture were performed, which is standard in aDNA studies [99], according to manufacturer’s instructions. The enriched libraries were sequenced on the Illumina HiSeq 6000 (2x100).

Line 509: For the M. leprae genome mapping, what parameters were used? Was seed disabled? Was -n still kept at 0.01 (as in case of the human DNA mapping), or was it increased to 0.1 (which is typically done in ancient microbial studies)?

The same parameters were used for human and pathogen mapping (-n 0.01 -l 300). The setting -n 0.01 is very stringent, allowing only 1% mismatches in the seed region. This reduces sensitivity (some authentic reads may fail to align if they contain damage or sequencing errors) but increases specificity by minimizing spurious alignments. By contrast, -n 0.1, which is often used in ancient microbial studies, would allow more mismatches and therefore capture more degraded reads at the cost of potentially introducing false positives. In the case of M. leprae, where metagenomic data may include multiple environmental Mycobacterium species from soil or other sources, using a stringent setting (-n 0.01) is appropriate to ensure high-confidence mapping.

Lines 512-517: Based on the variant calling parameters used and the statistics given in Table 3, strains G91 and G35 really do not have sufficient 3-fold genome coverage to be included in a tree. I think it is fine to say you recovered partial genomes, but given that only 7% and 20% of the genome, respectively, is callable for those two strains, you are incorporating a lot of missing data. At the very least, the authors should exclude those two strains from the tree and see if they get the same topology.

For the phylogenetic analysis, a set of previously published 3124 informative SNPs was first extracted from the VCF files. The minimum coverage of 3x applies to these SNPs, not the entire *M. leprae* genome.

Lines 572-574: A set of 3124 phylogenetically informative SNPs [15] was extracted from the VCF (min. coverage 3x, min quality 30, majority call 90%).

The number of SNPs fulfilling all filtering criteria for strains G91 and G35 were 1893 (60%) and 637 (20%), respectively. Phylogenetic analysis was performed again with 100 bootstrap replicates excluding these two strains to assess whether their inclusion affects the tree topology. When strains G91 and G35 were excluded, the topology of the tree remained unaffected (see Figure 2 below).

Figure 2: A maximum-likelihood tree illustrating the phylogenetic position of the *M. leprae* strains with the exclusion of Melaten G91 and G35 strains. Bootstrap support (100 bootstrap replicates) is marked for each node.

Information about the relatively low coverage of G91 and G35 was added to the results section for increased clarity.

Lines 151-153: As the genomes of G91 and G35 were only partially reconstructed (60% and 20% of the informative SNPs were covered), the placement of these strains within the phylogenetic tree should be interpreted with caution.

**Minor edits:**

Line 58: Just a suggestion - change "characteristic" to "diagnostic".

The suggestion was incorporated into the manuscript (now line #60)

Line 88: Change to "samples".

The correction was made (now line #90)

Line 146: The words "As an aside" seem weird/incorrect to use in this type of writing.

Maybe use "Furthermore" instead?

We accepted the suggestion and changed it to “Furthermore” (line #146)

Line 161: Typo - The word "three" is used instead of "tree".
The typo was corrected (now line #164)

Line 330: Change "the European Middle Ages" to "medieval Europe" or "the Middle Ages

in Europe".

We now use the term “medieval Europe” (now line #356)

Line 357: Spell out MS as multiple sclerosis since it is the first time it is used in the

manuscript.

The correction was made (now lines 382-383)

Line 483" Typo - It should be "UDG", not "UGD".
The typo was corrected.

Lines 480-485: No paper is cited to show which DNA extraction protocol was used. If this is the same as Krause-Kyora et al. 2018, this paper should be cited. Secondly, as far as I can tell, there was no half-UDG treatment done in Krause-Kyora et al. 2018 - it seems to be the libraries in that study were either full-UDG treated or non-UDG treated. Please check the correct citation for your half-UDG treatment protocol.

We have now added appropriate citations for both extraction and half UDG protocols (now line # 519).

Lines 503-508: Please change the sentence to remove the word "blasted". BLAST should be capitalized and not used as a verb. The sentence should read "The aligned sequences were then used as queries in a MegaBLAST search against the core nucleotide database (core_nt) on the NCBI website and the leprosy-positive status was assigned when the BLAST analysis revealed sequences unique to M. leprae."

The description of the method was updated.

Lines 550-555: Reads that aligned specifically to the *M. leprae* reference after competitive mapping were manually inspected in Integrative Genomics Viewer (IGV) v2.17.1 [97]. Alignments were checked for even read distribution, absence of stacking, and lack of excessive SNPs or insertions. The identity of these reads was further confirmed with BLAST v2.15.0 [98], using a significance threshold of E-value lower than 1e-5. Only those samples that contained at least 5 *M. leprae* reads in competitive mapping were considered as positive for the pathogen’s DNA.

**Third round of review**

**Comments by the Editorial Board member**

We ask that you further edit your discussion related to ‘Addressing the limitations and potential of case-control association studies in past populations’ to fully address previous concerns by Referee 3, i.e. differentiate between asymptomatic infection vs asymptomatic carriage and the implications for the interpretation of the findings of your paper.

**Authors Response**

Thank you for the opportunity to revise our manuscript “Ancient DNA study provides

clues to leprosy susceptibility in medieval Europe.” We appreciate the constructive

guidance from the Editorial Board and Referee 3.

To address the remaining scientific concern, we expanded the section "Addressing the

limitations and potential of case–control association studies in past populations". The

new paragraph clarifies the distinction between asymptomatic systemic infection and

asymptomatic nasal carriage of M. leprae, explains that our screening strategy

primarily detects systemic, blood-borne infection, and outlines how this affects

interpretation of the HLA enrichment signals. We now make explicit that the case group

includes individuals with systemic infection of varying stages and that our findings most

directly relate to susceptibility to systemic infection rather than to clinical progression or

severity.

We have also implemented the requested editorial and data-related changes. All ENA

accessions are now live and cited in the required format. We have incorporated the

updated abstract, moved the former “Material” section into Methods, and revised the

structure, formatting, and language to follow Genome Biology guidelines.

Supplementary files have been renumbered and listed with titles and descriptions as

required. The "Authors’ Contributions" statement has been updated.

We are currently collecting the remaining signatures for the authorship change form

and will submit the completed document shortly.

We hope these revisions address all remaining concerns, and we thank you again for

your guidance and consideration.
